# DNA Barcoding Evaluation and Its Taxonomic Implications in the Recently Evolved Genus *Oberonia* Lindl. (Orchidaceae) in China

**DOI:** 10.3389/fpls.2016.01791

**Published:** 2016-12-05

**Authors:** Yuling Li, Yi Tong, Fuwu Xing

**Affiliations:** ^1^Key Laboratory of Plant Resources Conservation and Sustainable Utilization, Guangdong Provincial Key Laboratory of Applied Botany, South China Botanical Garden, Chinese Academy of SciencesGuangzhou, China; ^2^College of Life Sciences, University of Chinese Academy of SciencesBeijing, China; ^3^Shanghai Chenshan Plant Science Research Center, Chinese Academy of SciencesShanghai, China; ^4^Shanghai Key Laboratory of Plant Functional Genomics and Resources, Shanghai Chenshan Botanical GardenShanghai, China

**Keywords:** DNA barcoding, *Oberonia*, taxonomy, radiation, species identification, ITS

## Abstract

The orchid genus *Oberonia* Lindl., is a taxonomically complex genus characterized by recent species radiations and many closely related species. All *Oberonia* species are under conservation as listed in the CITES and the IUCN Red List Categories and Criteria. Given its difficulties in taxonomy and conservation status, *Oberonia* is an excellent model for developing DNA barcodes. Three analytical methods and five DNA barcoding regions (*rbcL, matK, trnH-psbA*, ITS, and ITS2) were evaluated on 127 individuals representing 40 species and 1 variety of *Oberonia* from China. All the three plastid candidates tested (*rbcL, matK*, and *trnH-psbA*) have a lower discriminatory power than the nuclear regions (ITS and ITS2), and ITS had the highest resolution rate (82.14%). Two to four combinations of these gene sets were not better than the ITS alone, but when considering modes of inheritance, *rbcL*+ITS and *matK*+ITS were the best barcodes for identifying *Oberonia* species. Furthermore, the present barcoding system has many new insights in the current *Oberonia* taxonomy, such as correcting species identification, resolving taxonomic uncertainties, and the underlying presence of new or cryptic species in a genus with a complex speciation history.

## Introduction

*Oberonia* Lindl. is a monophyletic genus (Tang G. D. et al., 2015) with its infrageneric classification still unclear with involving many closely related and recently radiated species. It comprises about 150–200 species centered in tropical Asia and extending to tropical Africa, NE Australia and the Pacific islands. There are 44 species and 2 varieties distributed in China (Su, 2000; Chen et al., 2009; Lin and Lin, 2009; Ormerod, 2010; Xu et al., 2010; Tian et al., 2013), including some taxonomically complex groups lacking clear species delimitations, such as *O. acaulis* Griff., *O. acaulis* var. *luchunensis* S. C. Chen and *O. gongshanensis* Ormerod; *O. arisanensis* Hayata, *O. delicata* Z. H. Tsi and S. C. Chen, *O. japonica* (Maxim.) Makino and *O. menghaiensis* S. C. Chen; *O. austro-yunnanensis* S. C. Chen and Z. H. Tsi and *O. jenkinsiana* Griff. ex Lindl.; *O. insularis* Hayata and *O. sinica* (S. C. Chen and K. Y. Lang) Ormerod, etc. All of these taxonomically complex groups show slightly differences in the morphology of their leaves or flowers, while in some cases, characters are overlaps between species. For example, *O. japonica* and *O. delicata* are morphologically similar in most aspects except for some differences in flower character. The former is characterized by sepals broader than petals (sepals 0.9 mm, petals 0.7 mm), while the latter has equal sepals and petals in width. However, flowers with sepals broader than petals (sepals 0.8 mm, petals 0.6 mm) were also found in the population of *O. delicata* from type location in Yunnan Province (LYL037). The overlapping characters among species make the discrimination and delineation of this genus more challenging. In addition, *Oberonia* species are vegetative consensus and have diminutive flowers that are not easily discernable by the naked eye, making their identification and taxonomy exceedingly difficult even for professional taxonomists. Thus, a tool such as DNA barcoding, to aid rapid and accurate identification of these species is critically needed.

*Oberonia* species in China are mostly distributed in Yunnan, Guangxi, Guangdong, and Hainan provinces which belong to the Indo-Burma hot spots. All of them are endangered and listed in CITES (Conventions on International Trade of Endangered Species of Fauna and Flora, http://www.cites.org/eng/disc/species.shtml) and the IUCN Red List categories and criteria (http://www.zhb.gov.cn/gkml/hbb/bgg/201309/t20130912_260061.htm.) Habitat loss and illegal collection in this region poses a great threat to species survival, particularly in the case of narrow endemic *Oberonia* species that confined to one location in Yunnan Province (Cardinale et al., 2012). Rapidly and correctly identifying *Oberonia* species in China could promote the monitoring of endangered taxa.

DNA barcoding is a relatively new tool for species identification (Hebert et al., 2003, 2004) and has been applied in many areas, such as taxonomy (Meier et al., 2006; Huang et al., 2013; Mutanen et al., 2015), the discovery of new or cryptic species (e.g., Burns et al., 2008; Karanovic, 2015; Saitoh et al., 2015), biodiversity assessment and conservation (e.g., Taberlet et al., 2012; Ji et al., 2013; Liu et al., 2014), and monitoring the illegal wildlife trades (e.g., Baker, 2008; Gathier et al., 2013). After more than 10 years development, many plastid regions and nuclear regions have been suggested as universal barcodes for land plants, such as *rbcL*+*matK, trnH-psbA*, ITS, ITS2, etc. (CBOL Plant Working Group, 2009; Chen et al., 2010; Ren et al., 2010; Li et al., 2011). Although, significant progress has been made in the DNA barcoding, the discrimination of closely related species in recently evolved genera such as *Oberonia* Lindl., Willows (*Salix* L.) and *Curcuma* L. (Twyford, 2014; Chen et al., 2015), still poses a great challenge. Hence, testing DNA barcodes in such taxonomically difficult *Oberonia* genus could help to further understand the potential of these barcodes. The establishment of an available barcoding system for *Oberonia* could also facilitate the taxonomy and conservation of these taxa.

Five DNA barcode regions were assessed (*rbcL, matK, psbA*-*trnH*, ITS, and ITS2) in 40 species and 1 variety of *Oberonia* obtained from China. The objectives of this study are to: test the effectiveness of suggested core DNA barcodes (*rbcL*+*matK*) in *Oberonia*; evaluate the resolution of these five barcodes and in 2- to 4-region combinations to correctly identify individuals and discriminate among closely related species; and explore some of the taxonomic implications in *Oberonia*.

## Materials and methods

### Taxon sampling, DNA extraction, amplification, sequencing, and sequence downloads

In this study, 127 sequences from 40 species and 1 variety were collected for DNA sequencing, in which 123 sequences representing 39 species and 1 variety were from 6 provinces (Guangdong, Guangxi, Fujian, Hainan, Yunnan, and Tibet) in China. Four sequences from 1 species in Genbank were also included. In order to cover the morphological variability and geographical ranges, more individuals (>7) of widespread species such as *O. caulescens* Lindl. and *O. ensiformis* (Sm.) Lindl. were sampled. To test the potential of DNA barcodes, individuals of the taxonomically complex groups (e.g., *O. acaulis, O. acaulis* var. *luchunensis*, and *O. gongshanensis*; *O. jenkinsiana* and *O. austro-yunnanensis; O. arisanensis, O. delicata, O. japonica*, and *O. menghaiensis*; *O. insularis* and *O. sinica*) were sampled as much as possible. Detailed information about the samples is included in Table S1. The voucher specimens are deposited in the herbarium of the South China Botanical Garden, Chinese Academy of Sciences, Guangzhou (IBSC).

Total DNA was isolated from fresh or silica-dried leaves using a modified CTAB method (Doyle and Doyle, 1987). Primers are listed in Table S2. The ITS2 sequence was derived from the ITS (ITS, including ITS1, 5.8 s and ITS2) data directly. Two primer pairs of *trnH-psbA* were used for amplification and sequencing. Polymerase chain reaction (PCR) was conducted in a reaction mix (30 μl) each containing 10–20 ng (1–2 μl) of template DNA, 15 μl of 2 × PCR mix (0.005 units/μl Taq DNA polymerase, 4 mM MgCl_2_, and 0.4 mM dNTPs, TIANGEN), 1 μl of each primer (10 μM) and 11–12 μl of ddH_2_O. The PCR program started with a 2 min pre-melt stage at 98°C, followed by 36 cycles of 10 s at 98°C, annealing at 51–54°C (51°C for *rbcL* and ITS, 52°C for *matK*, 54°C for both two primer pairs of *trnH*-*psbA*) for 30 s, followed by 50 s at 68°C, and a final 8 min extension at 68°C. The PCR products were run on 1% agarose gels to check the quality of the amplified DNA. Then, PCR products with high quality were sent to Invitrogen (Shanghai) for purification and sequencing from both directions to reduce sequencing error.

### Data analyses

Sequences for each marker were edited and assembled using SEQUENCHER 4.14 (GenCodes, Corp. Ann Arbor) and then manually adjusted using Bioedit v7.1.3.0 (Hall, 1999). To assess the barcoding resolution for all barcodes (*rbcL, matK*, ITS, ITS2, *trnH*-*psbA, rbcL*+*matK, rbcL*+ITS, *rbcL*+ITS2, *rbcL*+*trnH*-*psbA, matK*+ITS, *matK*+ITS2, *matK*+*trnH*-*psbA, rbcL*+*matK*+ITS, *rbcL*+*matK*+ITS2, *rbcL*+*matK*+*trnH*-*psbA, rbcL*+*matK*+ITS+*trnH*-*psbA*), three analytical methods were employed, i.e., the pair-wise genetic distance method (PWG-distance), the sequence similarity method (TAXONDNA) and a phylogenetic-based method (Neighbor-Joining trees and Bayesian inference trees).

For the pair-wise genetic-based method, six parameters [average inter-specific distance, average theta (Θ) prime and smallest inter-specific distance; average intra-specific distance, average theta (Θ) and largest intra-specific distance] were calculated in Mega7 using the Kimura two-parameter distance model (K2P), to explore the intra- and inter-species variation (Chen et al., 2010; Pang et al., 2013; Kumar et al., 2016) We considered discrimination to be successful if the minimum inter-species distance involving a species, represented by more than one individual was larger than its maximum intra-species distance.

The sequence similarity method used the proportion of correct identifications identified with TAXONDNA/ Species Identifier 1.8 program, to assess the potential of all markers for accurate species identification. The “Best Match” (BM) and “Best Close Match” (BCM) tests in TAXONDNA were run for species that were represented by more than one individual (Meier et al., 2006).

For the phylogenetic-based method, Neighbor Joining (NJ) trees of all markers were conducted in Mega7 with K2P model and Bayesian inference (BI) trees were conducted in MrBayes v. 3.1 (Huelsenbeck and Ronquist, 2001; Ronquist and Huelsenbeck, 2003). If all the individuals of one species were clustered into a monophyletic group with the support nodes above 70% (NJ) or 95% (BI), then the species was considered as successfully identified. Species with a single specimen were included, but lacked depth to calculate significance. *Dendrobium strongylanthum* Rchb. f. (KF177656, KF143723, KF177553, and GU339107) was the outgroup for the tree-based analysis (Tang G. D. et al., 2015; Xu et al., 2015).

## Results

### PCR amplification, sequencing, and genetic divergence

The characteristics of five DNA barcoding regions and their combinations are shown in Table 1. When evaluated separately, *rbcL, matK*, and ITS had very high success rates (100%) for PCR amplification and sequencing using a single primer pair. Although, *trnH*-*psbA* also exhibited relatively high amplification success of 95.12% with two primer pairs, only 7 samples (5.69%) were successfully amplified and sequenced using the commonly used primer pair *trnH*2/*psbA*F (Sang et al., 1997; Tate and Simpson, 2003). The remaining samples were, however, generated for *trnH*-*psbA* using another primer *trnH*(GUG)/*psbA* (Hamilton, 1999). Overall, the aligned length of the five markers ranged from 269 bp (ITS2) to 1187 bp (*rbcL*). A total of 486 new sequences were submitted to NCBI, which included 123 sequences of each *rbcL, matK*, and ITS, separately, and 117 sequences of *trnH*-*psbA*, were submitted to NCBI (Table S1). In addition, we downloaded 12 sequences of *O. mucronata* (D. Don) Ormerod and Seidenf. from NCBI, including 4 sequences each of *rbcL, matK*, and ITS, respectively (JN005593, JN005592, JN005591, JN005590; JN004531, JN004530, JN004529, JN004528; JN114637, JN114636, JN114635, and JN114634). In total, 127 accessions of *rbcL, matK*, and ITS representing 40 species and 1 variety and 117 accessions of *trnH*-*psbA* representing 39 species were obtained for further analysis.

**Table 1:** **Evaluation of five DNA markers and combinations of the markers**.

	**R**	**M**	**I**	**I2**	**T**	**R+M**	**R+I**	**M+I**	**R+M+I**	**R+M+I2**	**R+M+T**	**R+M+I+T**
Universality of primers	Yes	Yes	Yes	Yes	No	−	−	−	−	−	−	−
Rate of PCR success (%)	100	100	100	100	100	−	−	−	−	−	−	−
Rate of sequencing success (%)	100	100	100	100	95.12	−	−	−	−	−	−	−
Aligned length (bp)	1187	815	827	269	1001	2002	2014	1642	2829	2271	3003	3830
Average intra-distance (%)	0.07	0.25	0.45	0.57	0.82	0.14	0.22	0.35	0.23	0.19	0.27	0.31
Average inter-distance (%)	0.57	1.90	6.74	9.92	2.25	1.14	3.08	4.21	2.70	2.08	1.33	2.63
Average theta (Θ) (%)	0.06	0.18	0.24	0.31	0.70	0.11	0.13	0.21	0.14	0.13	0.21	0.22
Coalescent Depth (%)	0.09	0.30	0.38	0.50	0.91	0.17	0.21	0.33	0.23	0.21	0.30	0.32
Proportion of variable sites (%)	5.48	19.63	37.12	52.42	10.49	11.24	18.42	28.44	18.77	16.12	10.76	16.42
Proportion of parsimony sites (%)	3.96	15.34	30.35	41.26	7.29	8.34	14.50	22.90	14.56	12.07	7.69	12.30

*R, rbcL; M, matK; I, ITS; I2, ITS2; T, trnH-psbA*.

Among the five markers, ITS2 had the highest proportion of variable sites (52.42%) and parsimony-informative sites (41.26%), and *rbcL* had the lowest. *RbcL* showed the lowest intra-specific and inter-specific divergence as well, whilst *trnH*-*psbA* showed the highest intra-specific divergence (0.82%), followed by ITS2 (0.57%). However, the greatest inter-specific distance was found in ITS2 (9.92%), followed by ITS (6.74%).

The average intra- and inter-specific divergence in the 11 combinations varied from 0.14 to 0.45% and 1.00 to 4.21%, respectively (Table 1). The combinations of *matK*+*trnH*-*psbA* and *matK*+ITS showed the highest intra- and inter-specific genetic divergence, respectively. The core barcode *rbcL*+*matK* and the combination of *rbcL*+*trnH*-*psbA* had the lowest intra-specific genetic divergence, respectively (Table 1).

### DNA barcoding gap assessment

The relative distribution of K2P distances based on single barcodes and their combinations indicated that ITS and ITS2 had relatively clear barcoding gaps, while the remaining three tested candidate barcodes and their combinations had overlaps between their inter- and intraspecific distances (Figure S1).

### Species resolution of candidate barcodes

**Figure 1:**
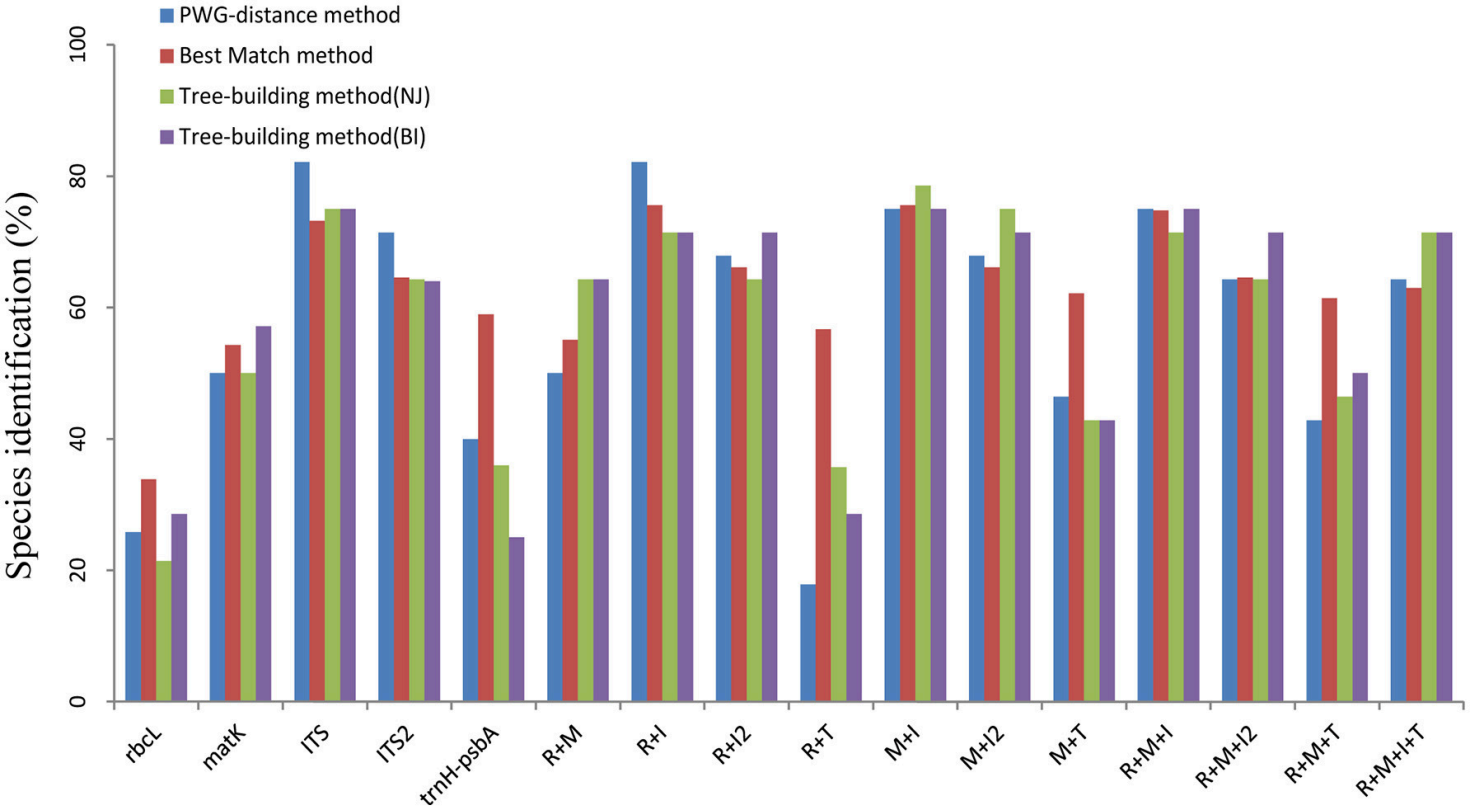
**Species discrimination rates of multiple candidate barcodes in *Oberonia***. R, *rbcL*; M, *matK*; I, ITS; I2, ITS2; T, *trnH*-*psbA*.

For the PWG-distance method, we used the local barcoding gap to reveal the species resolution power of candidate barcodes. That is, when a minimum inter-specific distance larger than the maximum intra-specific distance of a species, we considered it successfully identified. The proportion of the local barcoding gap varied among the markers tested (Figure S1 and Figure 1). Among single regions, the ITS exhibited the best species resolution (82.14%), followed by ITS2 (71.43%). In contrast, *rbcL* had the lowest species resolution (25.81%). Of the 11 combinations, *rbcL*+ITS showed the highest species resolution (82.14%), while *rbcL*+*trnH*-*psbA* showed the lowest species resolution (71.86%).

The TAXONDNA analysis based on BM and BCM methods exhibited similar discrimination success (Table 2). The ITS had the highest success rate for the correct identification of species (BM and BCM: 73.22%) among the five single barcodes, followed by the ITS2 (BM and BCM: 64.56%), whilst *rbcL* had the lowest resolution rate (BM and BCM: 33.85%). Among the 11 combinations, *rbcL*+ITS and *matK*+ITS performed the best (BM and BCM: 74.59%), followed by *rbcL*+*matK*+ITS (BM and BCM: 74.80%). However, the core barcode *rbcL*+*matK* demonstrated the lowest species resolution rate (BM and BCM: 55.11%).

**Table 2:** **Identification success based on the “best match” and “best close match” analysis methods**.

**Region**	**Best match**			**Best close match**		
	**Correct (%)**	**Ambiguous (%)**	**Incorrect (%)**	**Correct (%)**	**Ambiguous (%)**	**Incorrect (%)**
R	33.85	59.84	6.29	33.85	59.05	6.29
M	54.33	37.79	7.87	54.33	35.43	7.87
I	73.22	16.53	10.23	73.22	16.53	4.72
I2	64.56	26.77	8.66	64.56	26.77	4.72
T	58.97	20.51	20.51	58.97	20.51	20.51
R+M	55.11	30.7	14.17	55.11	29.92	12.59
R+I	75.59	12.59	11.81	75.59	12.59	7.87
R+I2	66.14	23.62	10.23	66.14	22.83	7.08
R+T	56.69	21.25	22.04	56.69	21.25	22.04
M+I	75.59	12.59	11.81	75.59	12.59	7.87
M+I2	66.14	23.62	10.23	66.14	22.04	8.66
M+T	62.2	14.17	23.62	62.2	14.17	23.62
R+M+I	74.8	12.59	12.59	74.8	12.59	8.66
R+M+I2	64.56	25.19	10.23	64.56	25.19	7.08
R+M+T	61.41	14.17	24.4	61.41	14.17	24.4
R+M+I+T	62.99	10.23	26.77	62.99	10.23	25.98

*R, rbcL; M, matK; I, ITS; I2, ITS2; T, trnH-psbA*.

The Neighbor Joining (NJ) and Bayesian inference (BI) tree-building method showed similar discriminatory results (Figure 1). Among the five single barcodes, the ITS demonstrated the best discrimination power (NJ tree and BI tree: 75%), followed by ITS2, while *rbcL* and *trnH-psbA* had the lowest discrimination power. In the 11 combination barcodes, *matK*+ITS reached the highest resolution power (NJ tree: 78.57%; BI tree: 75%), followed by *rbcL*+ITS, *rbcL*+*matK*+ITS, and *rbcL*+*matK*+ITS+*trnH-psbA*. The core barcode *rbcL*+*matK* recommended by COBL had poor resolution (NJ tree and BI tree: 64.29%), and only slightly better than all combinations with *trnH*-*psbA* (*rbcL*+*trnH*-*psbA, matK*+ *trnH*-*psbA*, and *rbcL*+*matK*+*trnH*-*psbA*).

## Discussion

### Universality of DNA barcodes

Primer universality is an important criterion for an ideal DNA barcode (Kress and Erickson, 2007). In this regard, the *rbcL, matK*, and ITS had the best performance in PCR amplification and sequencing among the four regions (successfully amplifying and sequencing 100% samples), consistent with many previous studies (Xu et al., 2015; Yan et al., 2015). However, compared to the above three barcodes, *trnH-psbA* had a relatively low sequencing success rate of 95.12% when two primer pairs were used. This was due largely to poly (T) tracts at about 100 bp in the forward direction when sequencing. In addition, a 230 bp indel in three sequences of two species, i.e., *O. intermedia* King and Pantl and *O. delicata* resulted in alignment difficulties. As for the insertion events, small inversions associated with palindromes, and sequencing problems related to mononucleotide repeats within this non-coding chloroplast region will complicate its use as a barcode (Whitlock et al., 2010). Thus, sequence alignment of this region must be careful to avoid overestimates of the substitution events.

### The resolution of tested candidate barcodes in *Oberonia*

When evaluated alone, the three plastid regions studied (*rbcL, matK, trnH*-*psbA*) had a resolution ranging from 21.43 to 57.14% (based on tree-building analysis), which is much lower than the discriminatory rate of nuclear region (Figure 1). Thus, all the single plastid regions are not recommended as DNA barcodes for the genus *Oberonia*. The low resolution of chloroplast regions has been previously reported in other plants including *Paphiopedilum* (25.74%), *Curcuma* (21.66%), and *Quercus* (0%) (Piredda et al., 2011; Chen et al., 2015; Guo et al., 2015). This could be due to the lower substitution rates that are found in plastid genomes, relative to their nuclear regions. Consequently, this highlights a need to search for nuclear DNA barcodes.

For the nuclear genome, the ITS and ITS2 generally provided better identification rates than the chloroplast sequences (Chen et al., 2010; Yao et al., 2010; Li et al., 2011). Likewise, the ITS and ITS2 have more parsimony informative sites, larger inter-specific distances and more discriminatory power than chloroplast regions in this study. In comparison the ITS distinguished 75% of monophyletic species, which was the best discrimination performance among the five loci (Figure 1). Meanwhile, any combinations with ITS produced better results than those combinations without (Figure 1 and Table 1). According to some previous results, it is difficult to amplify and directly sequence the region in some taxa because of incomplete concerted evolution of this multiple-copy region caused by hybridization or other factors (Alvarez and Wendel, 2003). However, it is not a problem in *Oberonia* and it has been widely used to generate phylogenies in many orchid taxa (Koehler et al., 2002; Zhai et al., 2014; Tang Y. et al., 2015). The amplification and sequencing rates of the ITS in this study were nearly 100%. Overall, for a single barcode, the ITS is the best candidate for *Oberonia*. Meanwhile, our results indicate that the ITS2 alone or in combinations with plastid markers did not have a higher discriminatory power than the ITS and/or its corresponding combinations (Figure 1 and Table 1). However, considering the ease of amplification, we suggest that the ITS2 may be an ideal supplementary barcode when ITS amplification has failed.

**Figure 2:**
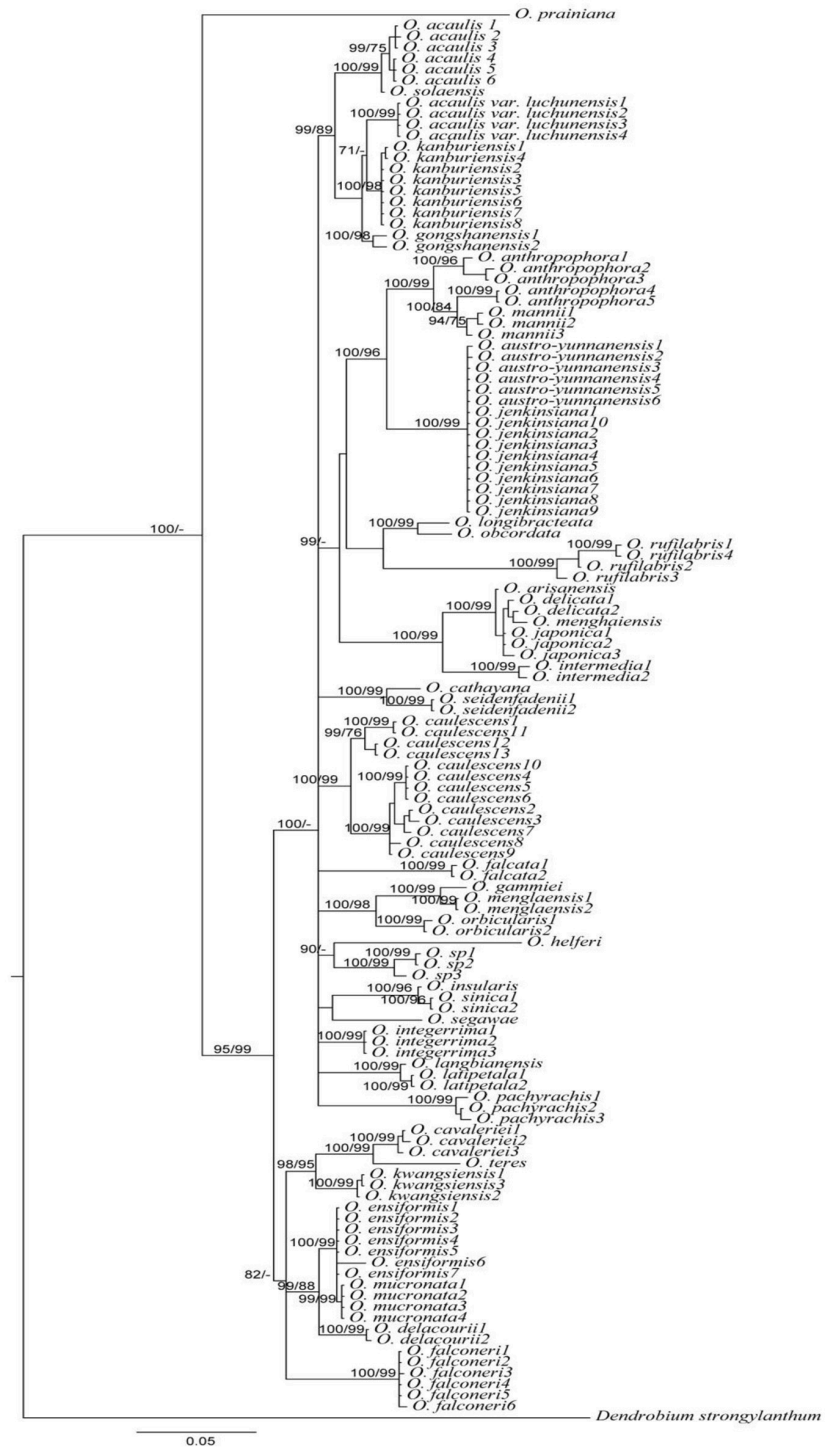
**Bayesian inference (BI) tree based on the combination of *matK*+ITS sequences in *Oberonia***. Numbers on branches represent BI and NJ support values, respectively.

Multi-locus barcodes have been suggested as DNA barcodes for land plants and can often improve the resolution rate of species identification (CBOL Plant Working Group, 2009; Li et al., 2011). In this study, the two-locus barcode *rbcL*+*matK*, recommended by the CBOL Plant Working Group (2009), had a low discrimination rate of 64.29% based on the Tree-building method (Figure 1), which was lower than the identification rate of 72% proposed by the CBOL Plant Working Group (2009). One of the most plausible explanations for this discrepancy is that the CBOL Plant Working Group focused on assessing the relative, rather than the absolute discriminatory power of the tested barcodes. We sampled more closely related species within the *Oberonia* genus and while *rbcL* and *matK* discriminates among genera well, the resolution rates of these two markers, alone and in combination, decreased at infrageneric levels, especially within recently evolved genera. Of the 2- to 4- combinations of the five loci tested, *rbcL*+ITS and *matK*+ITS exhibited the best discriminatory performance, almost the same as the single ITS barcode (Figure 2, Figures S2, S3). In plant DNA barcoding studies, the use of markers from different genomes with different modes of inheritance has been suggested, because such combinations of DNA markers will further our understanding of species delimitation and the evolutionary processes of speciation (Li et al., 2011). Although the resolution rates of *rbcL*+ITS and *matK*+ITS were not better than the single marker ITS itself, we suggest that the combination marker either *rbcL*+ITS or *matK*+ITS, should be the first choice to barcode *Oberonia* species.

### Implications of DNA barcoding for the current taxonomy of *Oberonia*

The results obtained in this study shed some light on the identification and taxonomy of the genus *Oberonia*. In previous DNA barcoding studies on a single genus or between closely related groups, species misidentification can be corrected with DNA barcoding (Pryer et al., 2010; Zhang et al., 2012; Yan et al., 2015). In the present study, the sample *O. kanburiensis* 1 was initially identified as *O. acaulis* Griff., based on leaf morphology (lacked flowers). DNA barcoding showed that the sequences of this sample were different from the other samples of this species, and it continually clustered with *O. kanburiensis* Seidenf. sequences. Confusion often occurs between *O. acaulis* and *O. kanburiensis*, because their morphology is similar prior to flowering. After blooming we rechecked the specimen and confirmed that this accession was misidentified and was in-fact *O. kanburiensis*. Such misidentifications were also found in *O. delacourii* 1 and *O. delacourii* 2 which were initially identified as *O. ensiformis* (Smith) Lindl. based on leaf morphology (lacked flowers). This finding indicates that DNA barcoding can differentiate species, with small sample input, great speed, and higher reliability, than previous methods. Thus, a more robust method has been demonstrated for endangered species monitoring in *Oberonia* genus.

In addition to the effectiveness of correcting misidentified specimen, DNA barcoding could also help in resolving taxonomic uncertainties. For example, *O. austro-yunnanensis* S. C. Chen et Z. H. Tsi and *O. jenkinsiana* Griff. ex Lindl. were considered as two separate species, discriminated by a joint at the base of the leaf (*O. austro-yunnanensis* with a basal joint vs. *O. jenkinsiana* without a basal joint) and subtle differences in the lip of flowers. After carefully checking the specimen and the original literature, we found that neither *O. austro-yunnanensis* nor *O. jenkinsiana* have joints at the base of the leaf, and are morphologically similar in most respects. We suspect that these are the same species. In the phylogenetic tree (Figure 2) *O. austro-yunnanensis* continually clustered with *O. jenkinsiana*. Besides, *O. jenkinsiana* is a widespread species and the geographical distribution ranges of these two species overlap. Therefore, our analysis implies that species with identical morphology, distribution, and sequences should be treated as one species. It is also probable that *O. sinica* and *O. insularis* reflect a similar situation. In another situation, *Oberonia acaulis* var. *luchunensis* are treated as a variety of *O. acaulis*, but in the light of these DNA barcoding results and the large difference in morphology, *O. acaulis* var. *luchunensis* could be raised to an independent species rather than a variety of *O. acaulis*, although further work is necessary (Figure 2).

Discovery of new or cryptic species is an important application of DNA barcoding within taxonomy (DeSalle et al., 2005). Many studies have employed the DNA barcoding to discover new or cryptic species in a broad range of animals and plants (Burns et al., 2008; Liu et al., 2011; Saitoh et al., 2015). In our study, some taxa such as samples *Oberonia* sp. 1, 2, 3 are similar to *Oberonia caulescens* Lindl. with subtle morphological differences in flowers and leaves, not withholding geographical divergences. However, they did not cluster with *O. caulescens* in the phylogenetic trees, indicating the existence of a new or cryptic species, yet additional study is necessary.

Morphological characterization associated with geographical, ecological, reproductive, and molecular data will facilitate the construction of a robust taxonomic system for any particular taxa. However, taxonomy and species delimitation within a single genus, especially genera with closely related species, is more difficult. In our study, despite the excellent performance of DNA barcoding in the *Oberonia* species from China, DNA barcoding does have difficulties in discriminating closely related species. For example, taxonomic complex group *O. arisanensis, O. delicata, O. japonica*, and *O. menghaiensis* are morphological similar with subtle differences in the diagnostic characteristic of their flowers. DNA barcoding did not discriminate these four species and they all clustered together in the *rbcL*+ITS tree and *matK*+ITS tree (Figure 2, Figures S2, S3). Another case for DNA barcoding failure occurred in *O. anthropophora*, which displayed incongruent signals of nuclear and plastid gene regions (Figures S2, S3). The slow rate of molecular evolution, paralogy, incidence of hybridization, introgression, and incomplete sorting of ancestral polymorphisms are the most likely sources of DNA barcoding failure for closely related *Oberonia* species (Funk and Omland, 2003; Hollingsworth et al., 2011; León-Romero et al., 2012). The exploration of more molecular markers, such as SNP and SSR, are needed to develop DNA barcodes to assist in species identification (Liu et al., 2008; Yuan et al., 2012; Zeng et al., 2012). This data will promote progress in DNA barcoding, while also facilitate the identification of endangered species.

## Author contributions

Designed the study: YL, YT, and FX. Performed the experiment and analyzed the data: YL and YT. Wrote the paper: YL, YT, and FX. All authors read and approved the final manuscript.

## Funding

This study was financially supported by the National Natural Science Foundation of China (Grant no. 31370231).

### Conflict of interest statement

The authors declare that the research was conducted in the absence of any commercial or financial relationships that could be construed as a potential conflict of interest.
