# Supplementary material for: DNA Barcoding Evaluation and Its Taxonomic Implications in the Recently Evolved Genus Oberonia Lindl. (Orchidaceae) in China
Source: Front Plant Sci. 2016 Dec 5;7:1791. doi: 10.3389/fpls.2016.01791 (PMC5136562; doi:10.3389/fpls.2016.01791)
Supplement: Supplementary file 1 [file DataSheet1.docx]

***Supplementary Material***

**Barcoding Evaluation and Its Taxonomic Implications in the Recently Evolved Genus *Oberonia* Lindl. (Orchidaceae) in China**

**Yuling Li^1, 2,^, Yi Tong^3, 4^, Fuwu Xing^1*^**

^1^Key Laboratory of Plant Resources Conservation and Sustainable Utilization, Guangdong Provincial Key Laboratory of Applied Botany, South China Botanical Garden, Chinese Academy of Sciences, Guangzhou, China,

^2^College of Life Sciences, University of Chinese Academy of Sciences, Beijing, China,

^4^Shanghai Chenshan Plant Science Research Center, Chinese Academy of Sciences, Shanghai, China,

^5^Shanghai Key Laboratory of Plant Functional Genomics and Resources, Shanghai Chenshan Botanical Garden, Shanghai, China

*** Correspondence:** Fuwu Xing: [xinfw@scib.ac.cn](mailto:xinfw@scib.ac.cn)

# Supplementary Tables and Figures

**Table S1** Samples, vouchers information and Genbank Accession number of *Oberonia* species in this study. Taxon names were arranged alphabetically.

| Taxon | Voucher | ITS | *mat*K | *rbc*L | *trn*H-*psb*A |
| --- | --- | --- | --- | --- | --- |
| *O. acaulis* 1 | LYL001, Yunnan, China (IBSC) | KY242061 | KY241938 | KY241815 | KY242184 |
| *O. acaulis* 2 | LYL002, Yunnan, China (IBSC) | KY242062 | KY241939 | KY241816 | KY242185 |
| *O. acaulis* 3 | LYL003, Yunnan, China (IBSC) | KY242063 | KY241940 | KY241817 | KY242186 |
| *O. acaulis* 4 | LYL004, Yunnan, China (IBSC) | KY242064 | KY241941 | KY241818 | KY242187 |
| *O. acaulis* 5 | TY0093, Yunnan, China (IBSC) | KY242065 | KY241942 | KY241819 | KY242188 |
| *O. acaulis* 6 | LYL005, Yunnan, China (IBSC) | KY242066 | KY241943 | KY241820 | KY242189 |
| *O. acaulis* var. *luchunensis* 1 | LYL006, Yunnan, China (IBSC) | KY242067 | KY241944 | KY241821 | KY242190 |
| *O. acaulis* var. *luchunensis* 2 | LYL007, Yunnan, China (IBSC) | KY242068 | KY241945 | KY241822 | KY242191 |
| *O. acaulis* var. *luchunensis* 3 | LYL008, Yunnan, China (IBSC) | KY242069 | KY241946 | KY241823 | KY242192 |
| *O. acaulis* var. *luchunensis* 4 | LYL009, Yunnan, China (IBSC) | KY242070 | KY241947 | KY241824 | KY242193 |
| *O. anthropophora* 1 | LYL010, Yunnan, China (IBSC) | KY242071 | KY241948 | KY241825 | — |
| *O. anthropophora* 2 | LYL011, Hainan, China (IBSC) | KY242072 | KY241949 | KY241826 | KY242194 |
| *O. anthropophora* 3 | LYL012, Hainan, China (IBSC) | KY242073 | KY241950 | KY241827 | KY242195 |
| *O. anthropophora* 4 | LYL013, Yunnan, China (IBSC) | KY242074 | KY241951 | KY241828 | KY242196 |
| *O. anthropophora* 5 | LYL014, Yunnan, China (IBSC) | KY242075 | KY241952 | KY241829 | KY242197 |
| *O. arisanensis* | LYL097, Taiwan, China (IBSC) | KY242076 | KY241953 | KY241830 | KY242198 |
| *O. austro-yunnanensis* 1 | LYL015, Yunnan, China (IBSC) | KY242077 | KY241954 | KY241831 | KY242199 |
| *O. austro-yunnanensis* 2 | LYL016, Yunnan, China (IBSC) | KY242078 | KY241955 | KY241832 | KY242200 |
| *O. austro-yunnanensis* 3 | LYL017, Yunnan, China (IBSC) | KY242079 | KY241956 | KY241833 | KY242201 |
| *O. austro-yunnanensis* 4 | LYL018, Yunnan, China (IBSC) | KY242080 | KY241957 | KY241834 | KY242202 |
| *O. austro-yunnanensis* 5 | LYL019, Yunnan, China (IBSC) | KY242081 | KY241958 | KY241835 | KY242203 |
| *O. austro-yunnanensi*s 6 | LYL020, Yunnan, China (IBSC) | KY242082 | KY241959 | KY241836 | KY242204 |
| *O. cathayana* | LYL021, Guangxi, China (IBSC) | KY242083 | KY241960 | KY241837 | KY242205 |
| *O. caulescens* 1 | LYL022, Yunnan, China (IBSC) | KY242084 | KY241961 | KY241933 | KY242206 |
| *O. caulescens* 10 | LYL023, Yunnan, China (IBSC) | KY242085 | KY241962 | KY241838 | KY242207 |
| *O. caulescens* 11 | LYL024, Yunnan, China (IBSC) | KY242086 | KY241963 | KY241839 | KY242208 |
| *O. caulescens* 12 | LYL025, Yunnan, China (IBSC) | KY242087 | KY241964 | KY241840 | KY242209 |
| *O. caulescens* 13 | LYL026, Yunnan, China (IBSC) | KY242088 | KY241965 | KY241841 | KY242210 |
| *O. caulescens* 2 | LYL027, Yunnan, China (IBSC) | KY242089 | KY241966 | KY241842 | KY242211 |
| *O. caulescens* 3 | LYL028, Yunnan, China (IBSC) | KY242090 | KY241967 | KY241843 | KY242212 |
| *O. caulescens* 4 | LYL029, Yunnan, China (IBSC) | KY242091 | KY241968 | KY241844 | KY242213 |
| *O. caulescens* 5 | LYL030, Yunnan, China (IBSC) | KY242092 | KY241969 | KY241845 | KY242214 |
| *O. caulescens* 6 | LYL031, Yunnan, China (IBSC) | KY242093 | KY241970 | KY241846 | KY242215 |
| *O. caulescens* 7 | LYL032, Guangdong, China (IBSC) | KY242094 | KY241971 | KY241847 | KY242216 |
| *O. caulescens* 8 | LYL033, Guangdong, China (IBSC) | KY242095 | KY241972 | KY241848 | KY242217 |
| *O. caulescens* 9 | EM159, Yunnan, China (IBSC) | KY242096 | KY241973 | KY241849 | KY242218 |
| *O. cavaleriei* 1 | LYL034, Yunnan, China (IBSC) | KY242097 | KY241974 | KY241850 | KY242219 |
| *O. cavaleriei* 2 | LYL035, Yunnan, China (IBSC) | KY242098 | KY241975 | KY241851 | KY242220 |
| *O. cavaleriei* 3 | LYL036, Guangxi, China (IBSC) | KY242099 | KY241976 | KY241852 | KY242221 |
| *O. delacourii* 1 | LYL104, Guangxi, China (IBSC) | KY242182 | KY242059 | KY241932 | KY242300 |
| *O. delacourii* 2 | D51428, Guizhou, China (PE) | KY242183 | KY242060 | KY241937 | — |
| *O. delicata* 1 | LYL037, Yunnan, China (IBSC) | KY242100 | KY241977 | KY241853 | KY242222 |
| *O. delicata* 2 | LYL038, Yunnan, China (IBSC) | KY242101 | KY241978 | KY241854 | KY242223 |
| *O. ensiformis* 1 | LYL039, Yunnan, China (IBSC) | KY242102 | KY241979 | KY241855 | KY242224 |
| *O. ensiformis* 2 | TY0107, Yunnan, China (IBSC) | KY242103 | KY241980 | KY241856 | KY242225 |
| *O. ensiformis* 3 | LYL040, Yunnan, China (IBSC) | KY242104 | KY241981 | KY241857 | KY242226 |
| *O. ensiformis* 4 | TY0106, Yunnan, China (IBSC) | KY242105 | KY241982 | KY241858 | KY242227 |
| *O. ensiformis* 5 | TY0226, Yunnan, China (IBSC) | KY242106 | KY241983 | KY241859 | KY242228 |
| *O. ensiformis* 6 | LYL041, Yunnan, China (IBSC) | KY242107 | KY241984 | KY241860 | KY242229 |
| *O. ensiformis* 7 | LYL042, Yunnan, China (IBSC) | KY242108 | KY241985 | KY241861 | KY242230 |
| *O. falcata* 1 | THZ770, Yunnan, China (IBSC) | KY242109 | KY241986 | KY241862 | KY242231 |
| *O. falcata* 2 | LYL043, Yunnan, China (IBSC) | KY242110 | KY241987 | KY241863 | KY242232 |
| *O. falconeri* 1 | LYL044, Guangxi, China (IBSC) | KY242111 | KY241988 | KY241864 | KY242233 |
| *O. falconeri* 2 | LYL045, Yunnan, China (IBSC) | KY242112 | KY241989 | KY241865 | KY242234 |
| *O. falconeri* 3 | LYL046, Yunnan, China (IBSC) | KY242113 | KY241990 | KY241866 | KY242235 |
| *O. falconeri* 4 | LYL098 Hainan, China (IBSC) | KY242114 | KY241991 | KY241867 | KY242236 |
| *O. falconeri* 5 | LYL047, Hainan, China (IBSC) | KY242115 | KY241992 | KY241868 | KY242237 |
| *O. falconeri* 6 | LYL048, Hainan, China (IBSC) | KY242116 | KY241993 | KY241869 | KY242238 |
| *O. gammiei* | LYL049, Hainan, China (IBSC) | KY242117 | KY241994 | KY241870 | KY242239 |
| *O. gongshanensis* 1 | LYL050, Yunnan, China (IBSC) | KY242118 | KY241995 | KY241871 | KY242240 |
| *O. gongshanensis* 2 | LYL051, Yunnan, China (IBSC) | KY242119 | KY241996 | KY241872 | KY242241 |
| *O. helferi* | LYL052, Yunnan, China (IBSC) | KY242120 | KY241997 | KY241873 | KY242242 |
| *O. insularis* | LYL099, Taiwan, China (IBSC) | KY242121 | KY241998 | KY241934 | KY242243 |
| *O. integerrima* 1 | TY0185, Yunnan, China (IBSC) | KY242122 | KY241999 | KY241874 | KY242244 |
| *O. integerrima* 2 | LYL053, Yunnan, China (IBSC) | KY242123 | KY242000 | KY241875 | KY242245 |
| *O. integerrima* 3 | LYL054, Yunnan, China (IBSC) | KY242124 | KY242001 | KY241876 | KY242246 |
| *O. intermedia* 1 | LYL055, Hainan, China (IBSC) | KY242177 | KY242054 | KY241927 | — |
| *O. intermedia* 2 | LYL056, Hainan, China (IBSC) | KY242178 | KY242055 | KY241928 | KY242296 |
| *O. japonica* 1 | LYL057, Guangdong, China (IBSC) | KY242125 | KY242002 | KY241877 | — |
| *O. japonica* 2 | LYL058, Fujian, China (IBSC) | KY242126 | KY242003 | KY241878 | KY242247 |
| *O. japonica* 3 | LYL059, Hainan, China (IBSC) | KY242127 | KY242004 | KY241879 | KY242248 |
| *O. jenkinsiana* 1 | TY0130, Yunnan, China (IBSC) | KY242128 | KY242005 | KY241880 | KY242249 |
| *O. jenkinsiana* 10 | LYL060, Yunnan, China (IBSC) | KY242129 | KY242006 | KY241881 | KY242250 |
| *O. jenkinsiana* 2 | LYL061, Yunnan, China (IBSC) | KY242130 | KY242007 | KY241882 | KY242251 |
| *O. jenkinsiana* 3 | LYL062, Yunnan, China (IBSC) | KY242131 | KY242008 | KY241883 | KY242252 |
| *O. jenkinsiana* 4 | LYL063, Yunnan, China (IBSC) | KY242132 | KY242009 | KY241884 | KY242253 |
| *O. jenkinsiana* 5 | LYL064, Yunnan, China (IBSC) | KY242133 | KY242010 | KY241885 | KY242254 |
| *O. jenkinsiana* 6 | LYL065, Yunnan, China (IBSC) | KY242134 | KY242011 | KY241886 | KY242255 |
| *O. jenkinsiana* 7 | LYL066, Yunnan, China (IBSC) | KY242135 | KY242012 | KY241887 | KY242256 |
| *O. jenkinsiana* 8 | LYL067, Yunnan, China (IBSC) | KY242136 | KY242013 | KY241888 | KY242257 |
| *O. jenkinsiana* 9 | LYL068, Yunnan, China (IBSC) | KY242137 | KY242014 | KY241889 | KY242258 |
| *O. kanburiensis* 1 | TY0177, Yunnan, China (IBSC) | KY242138 | KY242015 | KY241890 | KY242259 |
| *O. kanburiensis* 2 | TY0075, Yunnan, China (IBSC) | KY242139 | KY242016 | KY241891 | — |
| *O. kanburiensis* 3 | THZ716, Yunnan, China (IBSC) | KY242140 | KY242017 | KY241892 | KY242260 |
| *O. kanburiensis* 4 | LYL069, Yunnan, China (IBSC) | KY242141 | KY242018 | KY241893 | KY242261 |
| *O. kanburiensis* 5 | LYL070, Yunnan, China (IBSC) | KY242142 | KY242019 | KY241894 | KY242262 |
| *O. kanburiensis* 6 | LYL071, Yunnan, China (IBSC) | KY242143 | KY242020 | KY241895 | KY242263 |
| *O. kanburiensis* 7 | LYL072, Yunnan, China (IBSC) | KY242144 | KY242021 | KY241896 | KY242264 |
| *O. kanburiensis* 8 | TY0236, Yunnan, China (IBSC) | KY242145 | KY242022 | KY241897 | KY242265 |
| *O. kwangsiensis* 1 | YLY100, Guangxi, China (IBSC) | KY242146 | KY242023 | KY241898 | KY242266 |
| *O. kwangsiensis* 2 | LYL106, Guangxi, China (IBSC) | KY242147 | KY242024 | KY241899 | KY242267 |
| *O. kwangsiensis* 3 | HMQ338, Guizhou, China (IBSC) | KY242148 | KY242025 | KY241900 | KY242268 |
| *O. langbianensis* | LYL073, Yunnan, China (IBSC) | KY242149 | KY242026 | KY241901 | KY242269 |
| *O. latipetala* 1 | LYL074, Yunnan, China (IBSC) | KY242150 | KY242027 | KY241902 | KY242270 |
| *O. latipetala* 2 | LYL075, Yunnan, China (IBSC) | KY242151 | KY242028 | KY241903 | KY242271 |
| *O. longibracteata* | LYL076, Hainan, China (IBSC) | KY242152 | KY242029 | KY241904 | KY242272 |
| *O. mannii* 1 | LYL077, Yunnan, China (IBSC) | KY242153 | KY242030 | KY241905 | KY242273 |
| *O. mannii* 2 | THZ759, Yunnan, China (IBSC) | KY242154 | KY242031 | KY241935 | KY242274 |
| *O. mannii* 3 | LYL078, Yunnan, China (IBSC) | KY242155 | KY242032 | KY241906 | KY242275 |
| *O. menghaiensis* | JXH10465, Yunnan, China (PE) | KY242156 | KY242033 | KY241907 | — |
| *O. menglaensis* 1 | LYL079, Hainan, China (IBSC) | KY242157 | KY242034 | KY241908 | KY242276 |
| *O. menglaensis* 2 | LYL080, Hainan, China (IBSC) | KY242158 | KY242035 | KY241909 | KY242277 |
| *O. orbicularis* 1 | TY0258, Yunnan, China (IBSC) | KY242179 | KY242056 | KY241929 | KY242297 |
| *O. orbicularis* 2 | LYL081, Yunnan, China (IBSC) | KY242180 | KY242057 | KY241930 | KY242298 |
| *O. obcordata* | LYL096, Tibet, China (IBSC) | KY242159 | KY242036 | KY241936 | KY242278 |
| *O. pachyrachis* 1 | LYL082, Yunnan, China (IBSC) | KY242160 | KY242037 | KY241910 | KY242279 |
| *O. pachyrachis* 2 | LYL083, Yunnan, China (IBSC) | KY242161 | KY242038 | KY241911 | KY242280 |
| *O. pachyrachis* 3 | LYL084, Yunnan, China (IBSC) | KY242162 | KY242039 | KY241912 | KY242281 |
| *O. prainiana* | LYL085, Yunnan, China (IBSC) | KY242163 | KY242040 | KY241913 | KY242282 |
| *O. rufilabris* 1 | LYL086, Hainan, China (IBSC) | KY242167 | KY242044 | KY241917 | KY242286 |
| *O. rufilabris* 2 | LYL087, Yunnan, China (IBSC) | KY242168 | KY242045 | KY241918 | KY242287 |
| *O. rufilabris* 3 | LYL103, Yunnan, China (IBSC) | KY242169 | KY242046 | KY241919 | KY242288 |
| *O. rufilabris* 4 | LYL088, Hainan, China (IBSC) | KY242170 | KY242047 | KY241920 | KY242289 |
| *O. segawae* | LYL089, Yunnan, China (IBSC) | KY242171 | KY242048 | KY241921 | KY242290 |
| *O. seidenfadenii* 1 | LYL105, Guangxi, China (IBSC) | KY242172 | KY242049 | KY241922 | KY242291 |
| *O. seidenfadenii* 2 | LYL435, Guangdong, China (IBSC) | KY242173 | KY242050 | KY241923 | KY242292 |
| *O. sinica* 1 | LYL090, Yunnan, China (IBSC) | KY242174 | KY242051 | KY241924 | KY242293 |
| *O. sinica* 2 | LYL091, Guangxi, China (IBSC) | KY242175 | KY242052 | KY241925 | KY242294 |
| *O. solaensis* | LYL092, Yunnan, China (IBSC) | KY242181 | KY242058 | KY241931 | KY242299 |
| *O. sp.* 1 | LYL093, Yunnan, China (IBSC) | KY242164 | KY242041 | KY241914 | KY242283 |
| *O. sp.* 2 | LYL094, Yunnan, China (IBSC) | KY242165 | KY242042 | KY241915 | KY242284 |
| *O. sp.* 3 | LYL101, Yunnan, China (IBSC) | KY242166 | KY242043 | KY241916 | KY242285 |
| *O. teres* | LYL095, Yunnan, China (IBSC) | KY242176 | KY242053 | KY241926 | KY242295 |

**Table S2** A list of primers used for PCR and sequence in this study.

| **Region primer sequence(5’-3’) reference** | | | |
| --- | --- | --- | --- |
| *rbcL* | 1F | ATG TCA CCA CAA ACA GAA AC | Fay et al., 1997 |
|  | 1360R | CTT CAC AAG CAG CAG CTA GTT C | Reeves et al., 2001 |
| *matK* | 390F | CGA TCT ATT CAT TCA ATA TTT C | Cuénoud et al., 2002 |
|  | 1326R | TCT AGC ACA CGA AAG TCG AAG T | Cuénoud et al., 2002 |
| ITS | 17SE | ACG AAT TCA TGG TCC GGT GAA GTG TTC G | Sun et al., 1994 |
|  | 26SE | TAG AAT TCC CCG GTT CGC TCG CCG TTA C | Sun et al., 1994 |
| *trnH*-*psbA* | *trnH*(GUG) | ACTGCCTTGATCCACTTGGC | Hamilton, 1999 |
|  | *psbA*  *trnH*2  *psbA*F | CGAAGCTCCATCTACAAATGG  CGC GCA TGG TGG ATT CAC AAT CC  GTT ATG CAT GAA CGT AAT GCT C | Hamilton, 1999  Tate & Simpson 2003  Sang et al., 1997 |

**Fig S1.** Intra- and Inter-specific distances using Kimura 2-parameter (K2P) model among five barcoding regions and their combinations. (A). *rbcL*, (B). *matK*, (C). ITS, (D). ITS2, (E). *trnH*-*psbA*, (F). *rbcL*+*matK*, (G). *rbcL*+ITS, (H). *rbcL*+ITS2, (I). *rbcL*+*trnH*-*psbA*, (J). *matK*+ITS, (K). *matK*+ITS2, (L). *matK*+*trnH*-*psbA*, (M). *rbcL*+*matK*+ITS, (N). *rbcL*+*matK*+ITS2, (O). *rbcL*+*matK*+*trnH*-*psbA*, (P). *rbcL*+*matK*+ITS+*trnH*-*psbA*

(A)

[键入文档的引述或关注点的摘要。您可将文本框放置在文档中的任何位置。请使用“绘图工具”选项卡更改引言文本框的格式。]

(B)

(C)

[键入文档的引述或关注点的摘要。您可将文本框放置在文档中的任何位置。请使用“绘图工具”选项卡更改引言文本框的格式。]

(D)

[键入文档的引述或关注点的摘要。您可将文本框放置在文档中的任何位置。请使用“绘图工具”选项卡更改引言文本框的格式。]

(F)

[键入文档的引述或关注点的摘要。您可将文本框放置在文档中的任何位置。请使用“绘图工具”选项卡更改引言文本框的格式。]

(E)

[键入文档的引述或关注点的摘要。您可将文本框放置在文档中的任何位置。请使用“绘图工具”选项卡更改引言文本框的格式。]

(H)

[键入文档的引述或关注点的摘要。您可将文本框放置在文档中的任何位置。请使用“绘图工具”选项卡更改引言文本框的格式。]

(G)

[键入文档的引述或关注点的摘要。您可将文本框放置在文档中的任何位置。请使用“绘图工具”选项卡更改引言文本框的格式。]

(J)

(I)

[键入文档的引述或关注点的摘要。您可将文本框放置在文档中的任何位置。请使用“绘图工具”选项卡更改引言文本框的格式。]

(K)

(L)

(N)

(M)

(O)

(P)

**Fig. S2** Neighour-joining tree with bootstrap support value above 70% for *Oberonia* generated using different regions and multiple combinations. (A) the *rbcL* region, (B) the *matK* region, (C) the *trnH*-*psbA* region, (D) the ITS region, (E) the ITS2 region, (F) the *rbcL*+*matK* combination, (G) the *rbcL*+ITS combination, (H) the *rbcL*+ITS2 combination, (I) the *rbcL*+*trnH*-*psbA* combination, (J) the *matK*+ITS combination, (K) the *matK*+ITS2 combination, (L) the *matK*+*trnH*-*psbA* combination, (M) the *rbcL*+*matK*+ITS combination (N) the *rbcL*+*matK*+ITS2 combination, (O) the *rbcL*+*matK*+*trnH*-*psbA* combination, (P) the *rbcL*+*matK*+ITS+*trnH*-*psbA* combination.

Fig. S2 (A): *rbcL*

Fig. S2 (B): *matK*

Fig. S2 (C): *trnH*-*psbA*

Fig. S2 (D): ITS

Fig. S2 (E): ITS2

Fig. S2 (F): *rbcL*+*matK*

Fig. S2 (G): *rbcL*+ITS

Fig. S2 (H): *rbcL*+ITS2

Fig. S2 (I): *rbcL+trnH-psbA*

Fig. S2 (J): *matK+*ITS

Fig. S2 (K): *matK+*ITS2

Fig. S2 (L): *matK+trnH*-*psbA*

Fig. S2 (M): *rbcL+matK+*ITS

Fig. S2 (N): *rbcL+matK+*ITS2

Fig. S2 (O): *rbcL+matK+trnH*-*psbA*

Fig. S2 (P): *rbcL+matK+*ITS*+trnH*-*psbA*

**Fig. S3** Bayesian inference tree with bootstrap support value above 70% for *Oberonia* generated using different regions and multiple combinations. (A) the *rbcL* region, (B) the *matK* region, (C) the *trnH*-*psbA* region, (D) the ITS region, (E) the ITS2 region, (F) the *rbcL*+*matK* combination, (G) the *rbcL*+ITS combination, (H) the *rbcL*+ITS2 combination, (I) the *rbcL*+*trnH*-*psbA* combination, (J) the *matK*+ITS combination, (K) the *matK*+ITS2 combination, (L) the *matK*+*trnH*-*psbA* combination, (M) the *rbcL*+*matK*+ITS combination (N) the *rbcL*+*matK*+ITS2 combination, (O) the *rbcL*+*matK*+*trnH*-*psbA* combination, (P) the *rbcL*+*matK*+ITS+*trnH*-*psbA* combination.


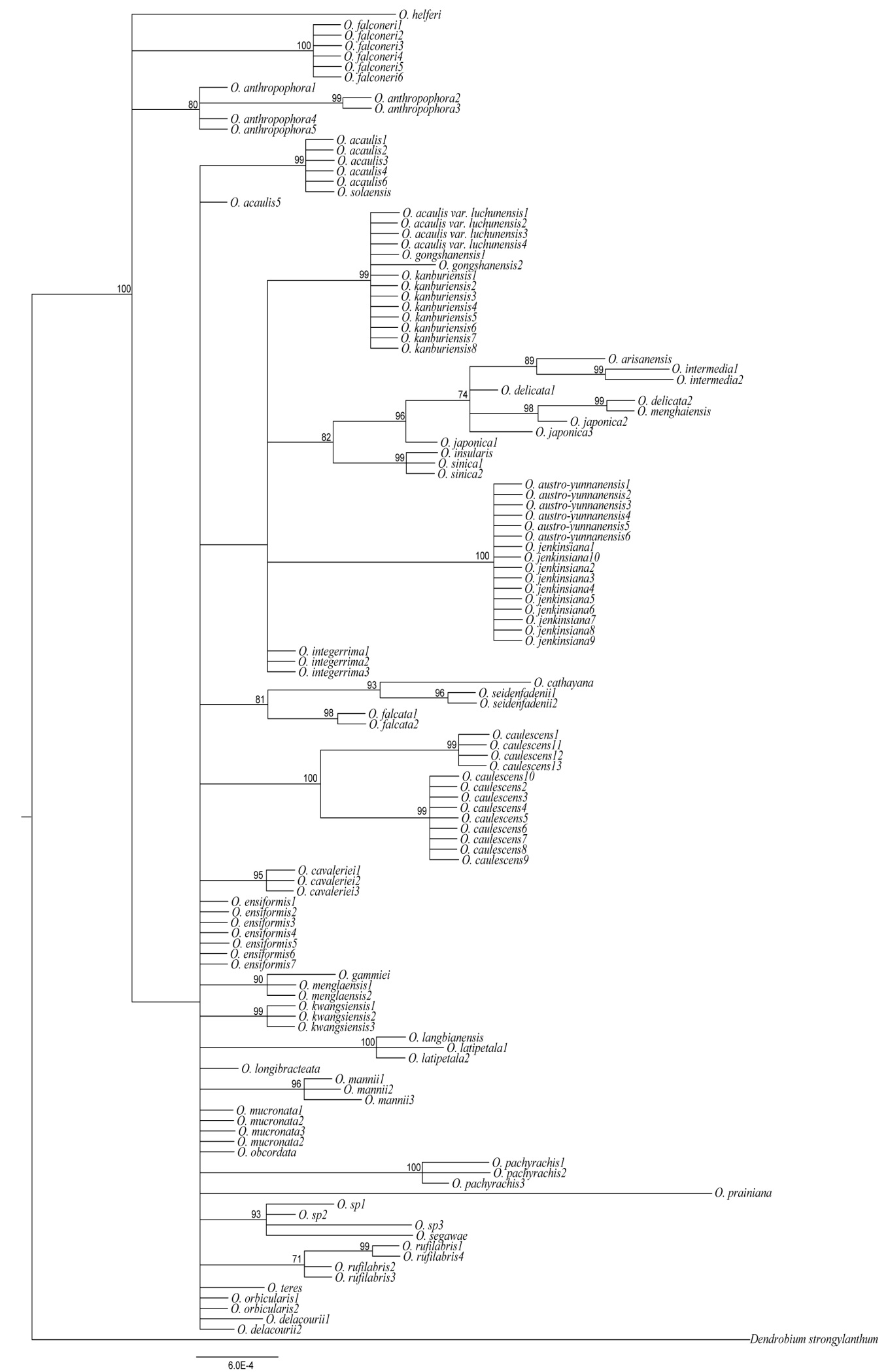


Fig. S3 (A): *rbcL*

rbcL


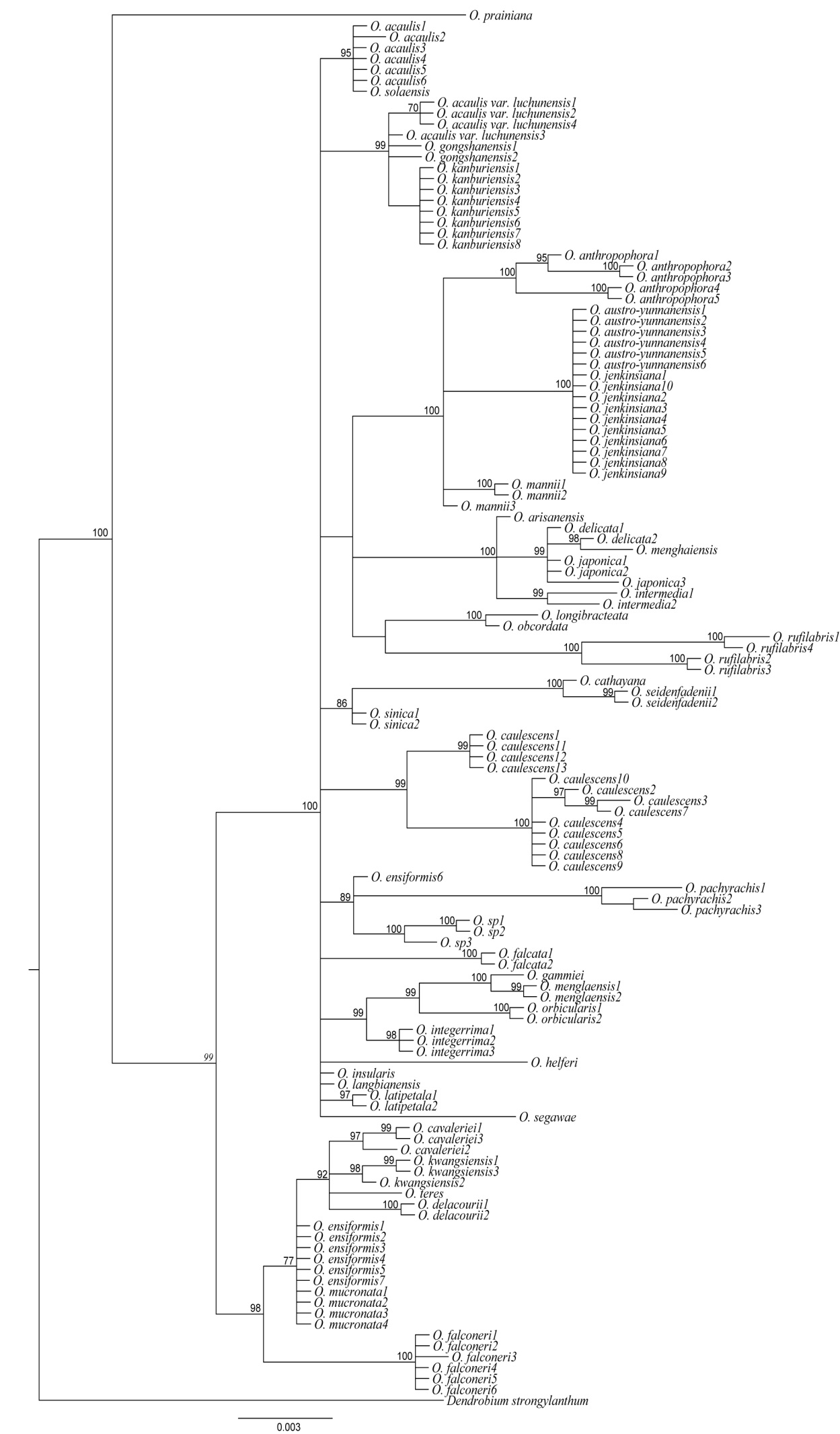


Fig. S3 (B): *matK*


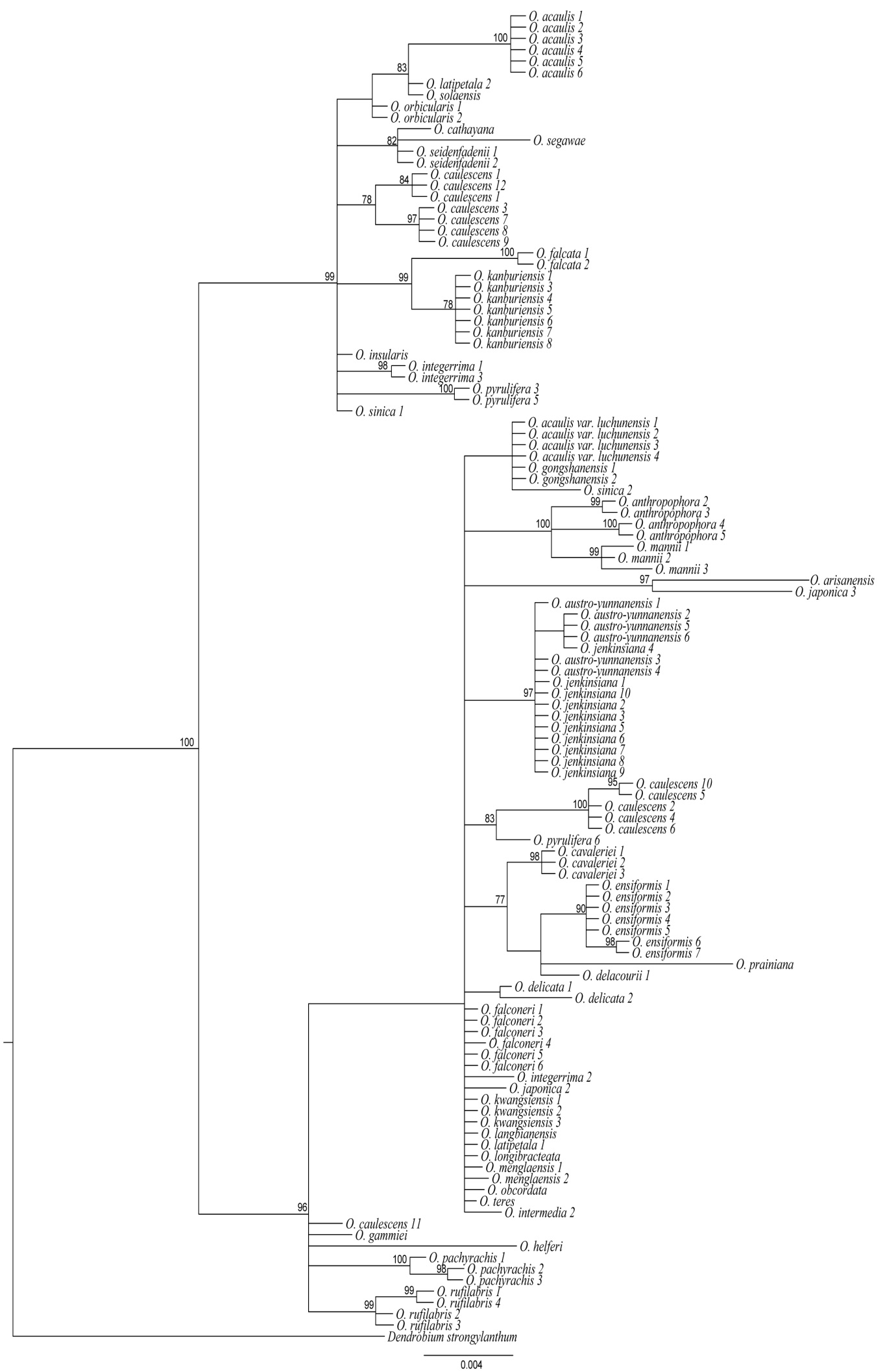

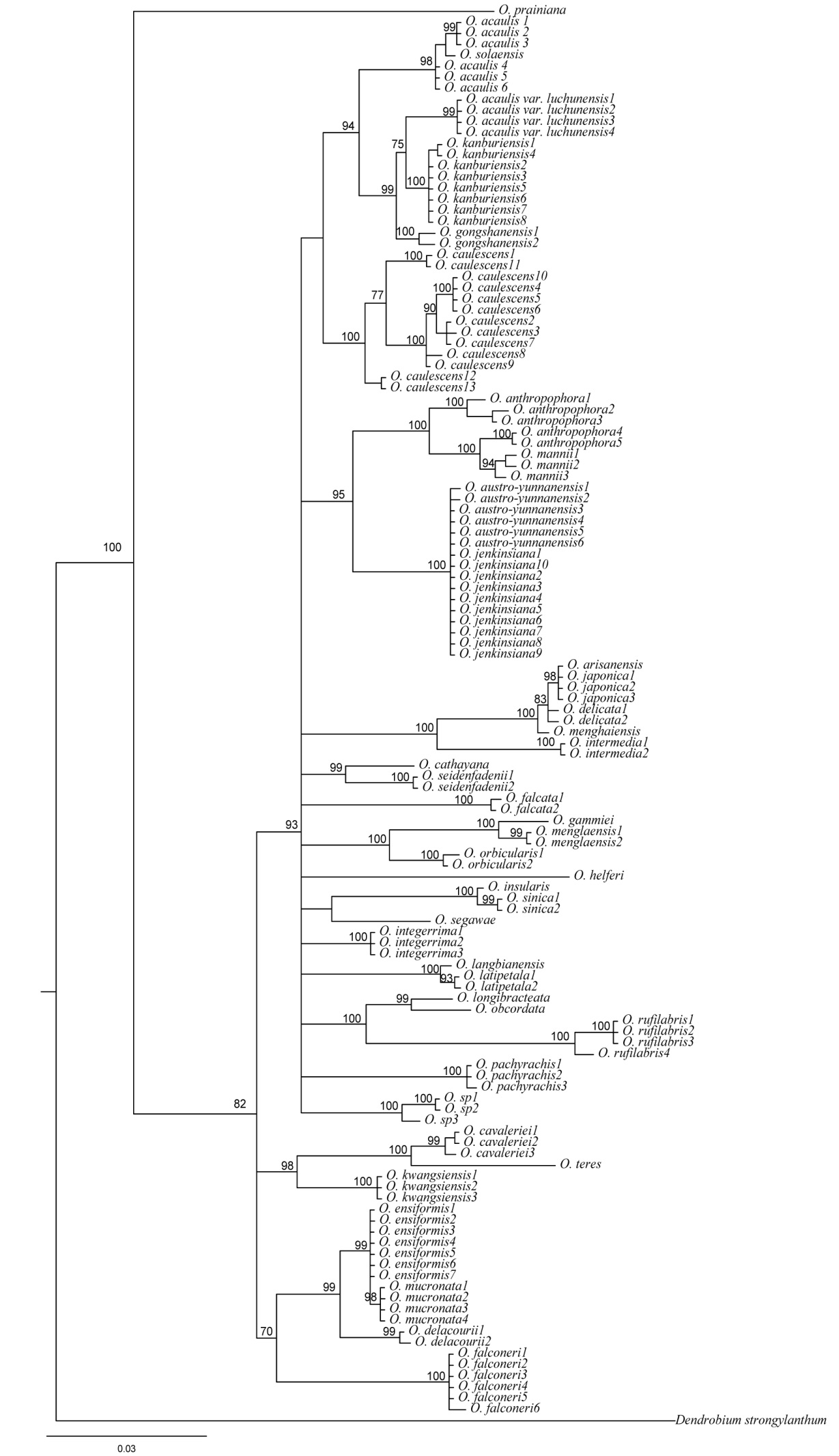


Fig. S3(C): *trnH*-*psbA*

Fig. S3 (D): ITS


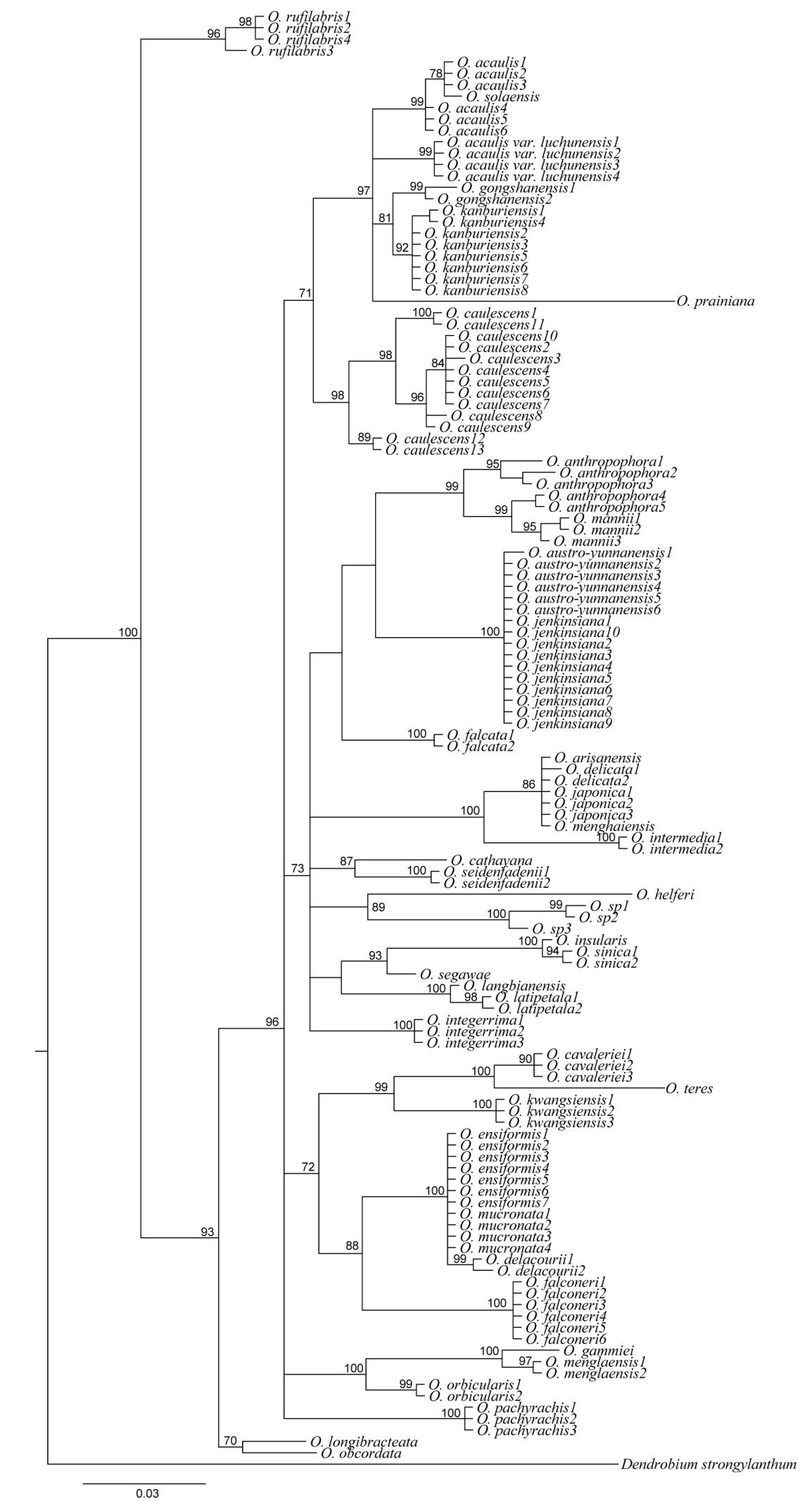


Fig. S3 (E): ITS2


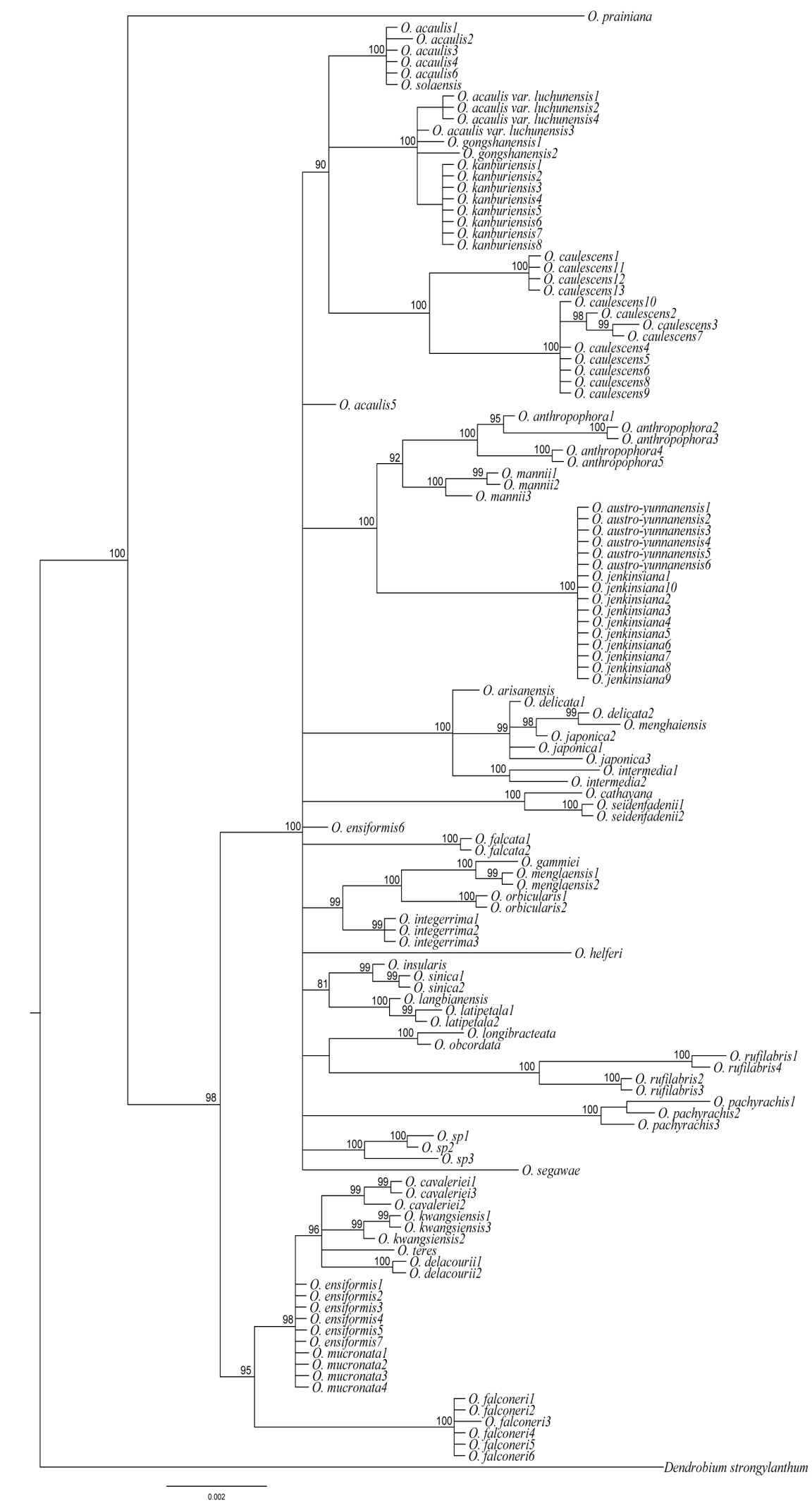


Fig. S3 (F): *rbcL*+*matK*


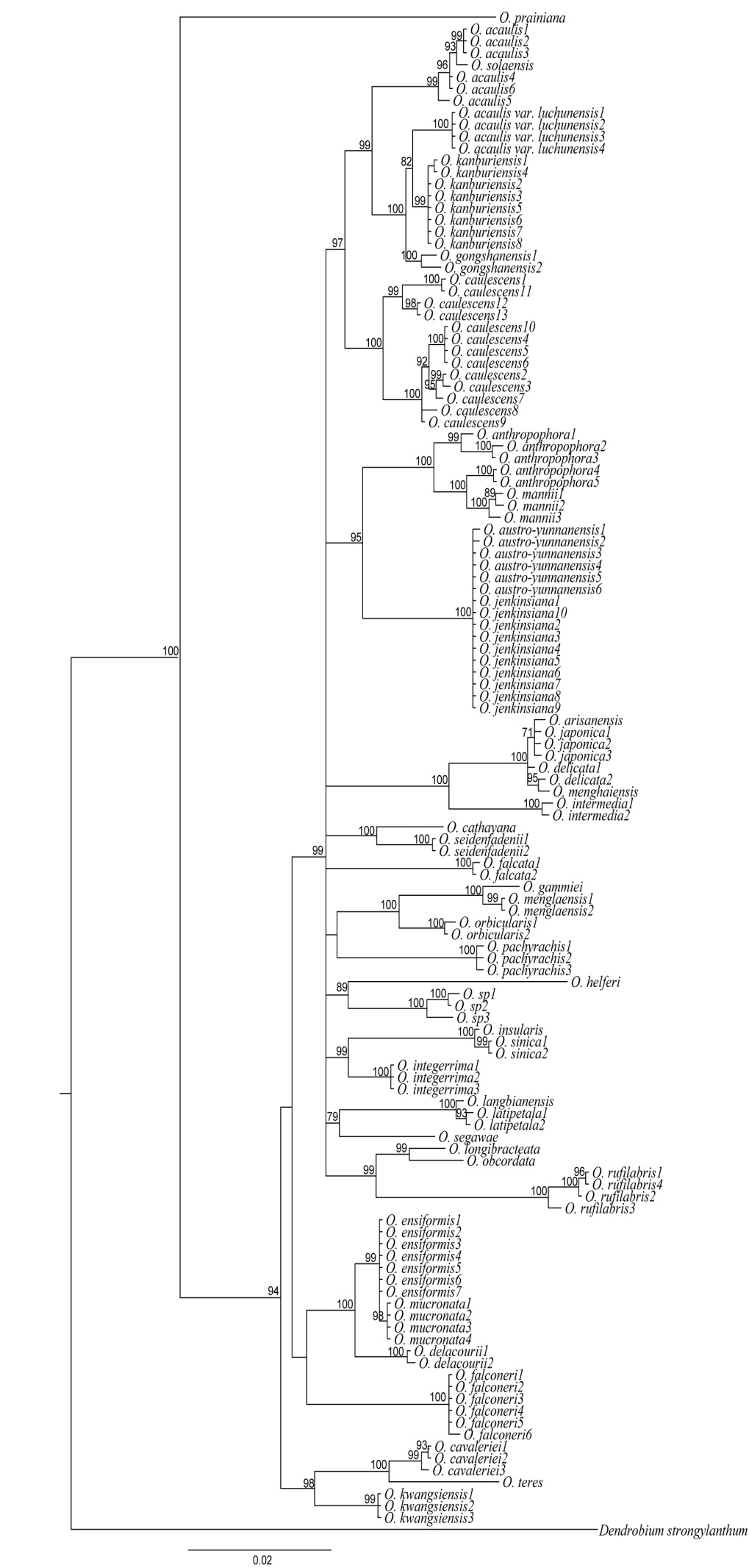


Fig. S3 (G): *rbcL*+ITS


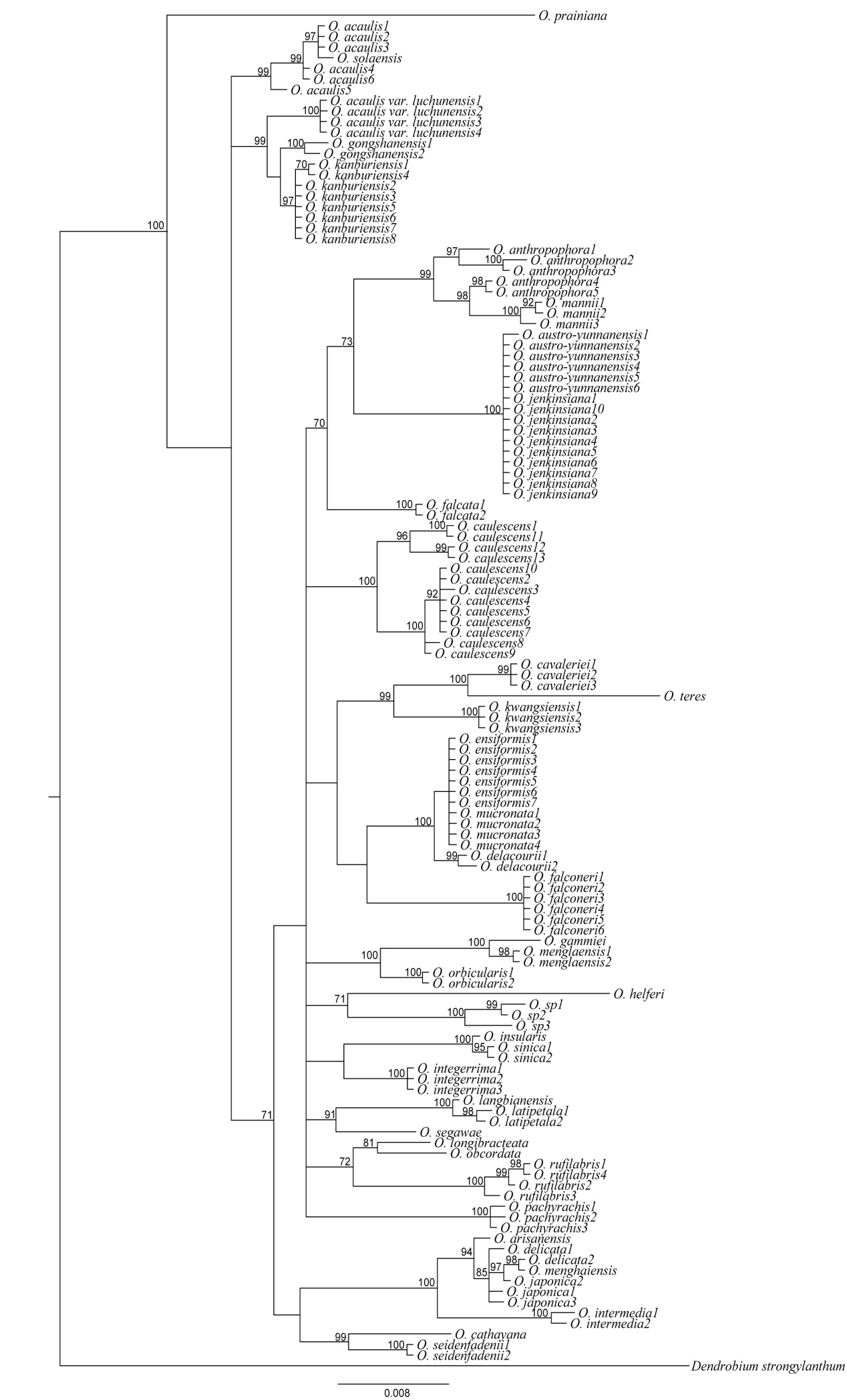


Fig. S3 (H): *rbcL*+ITS2


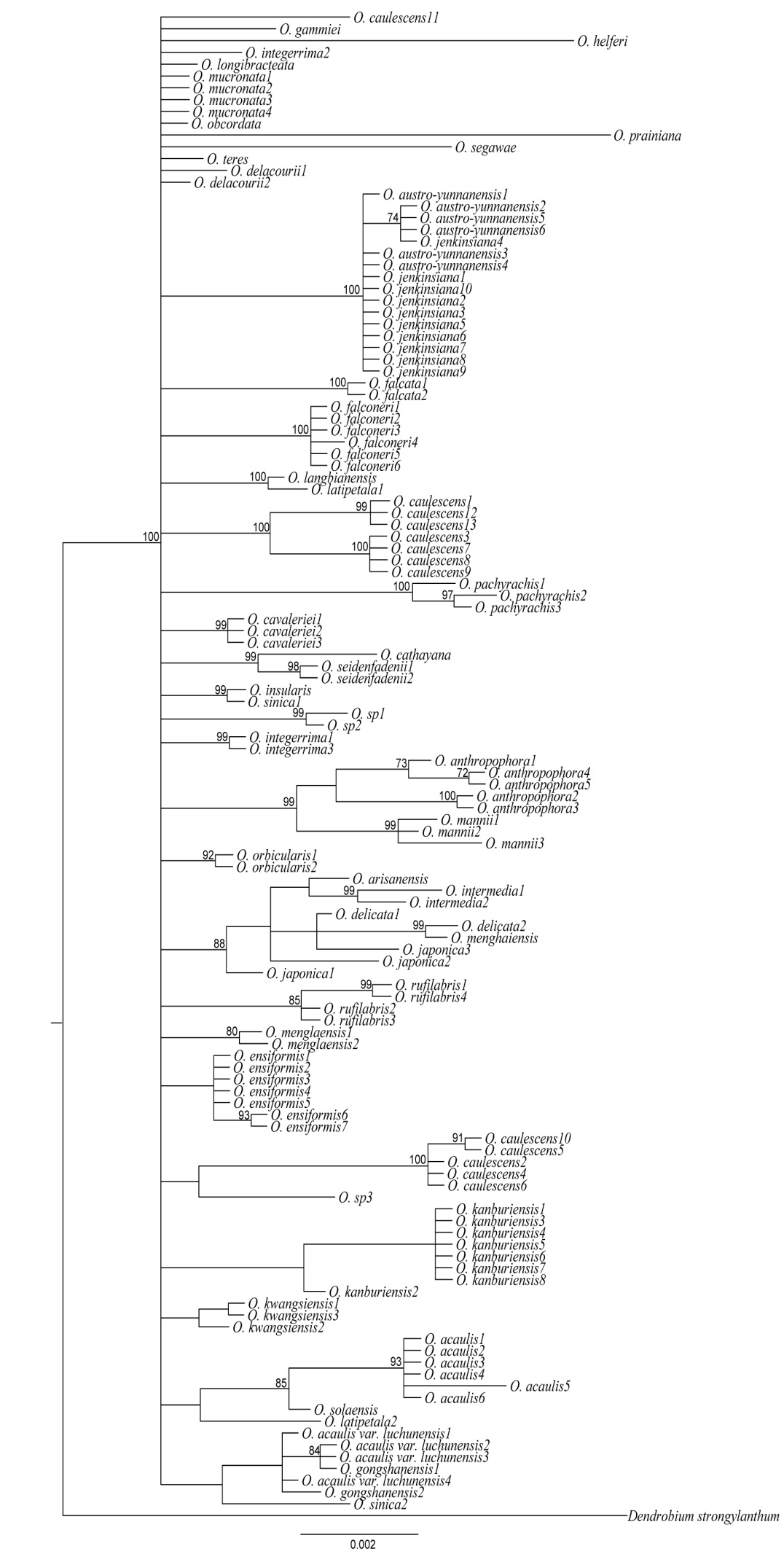


Fig. S3 (I): *rbcL+trnH-psbA*


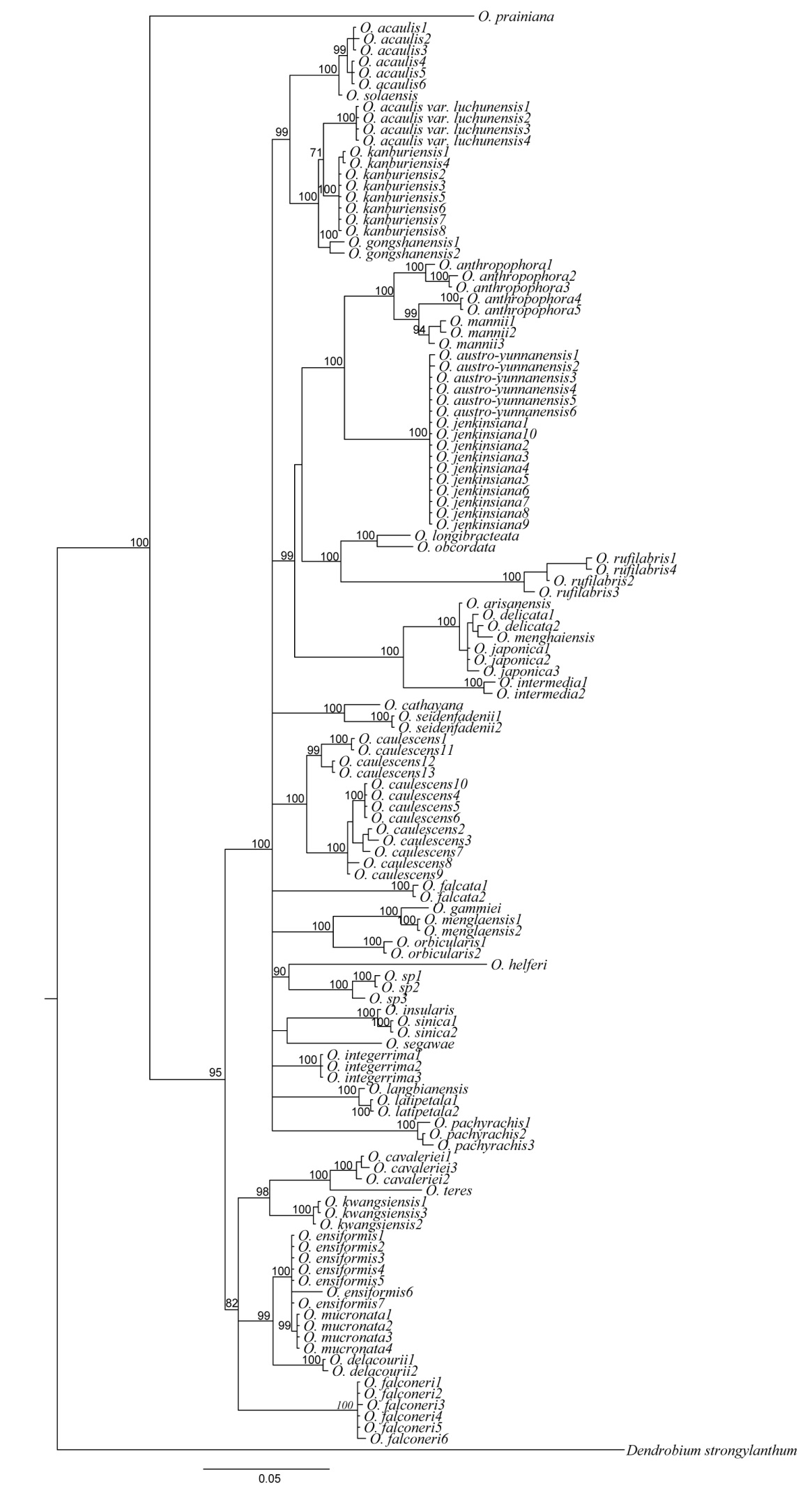


Fig. S3 (J): *matK+*ITS


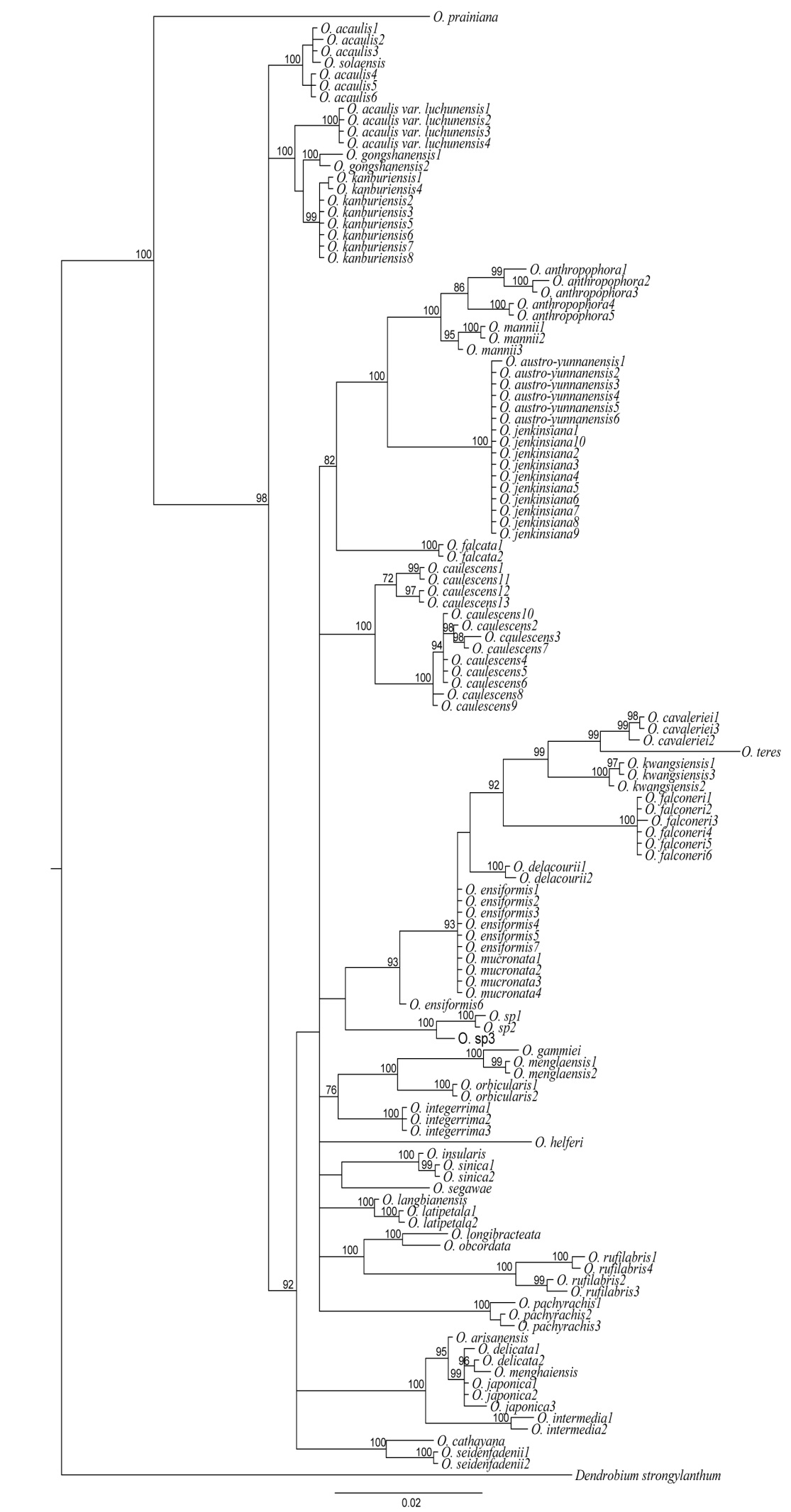


Fig. S3 (K): *matK+*ITS2


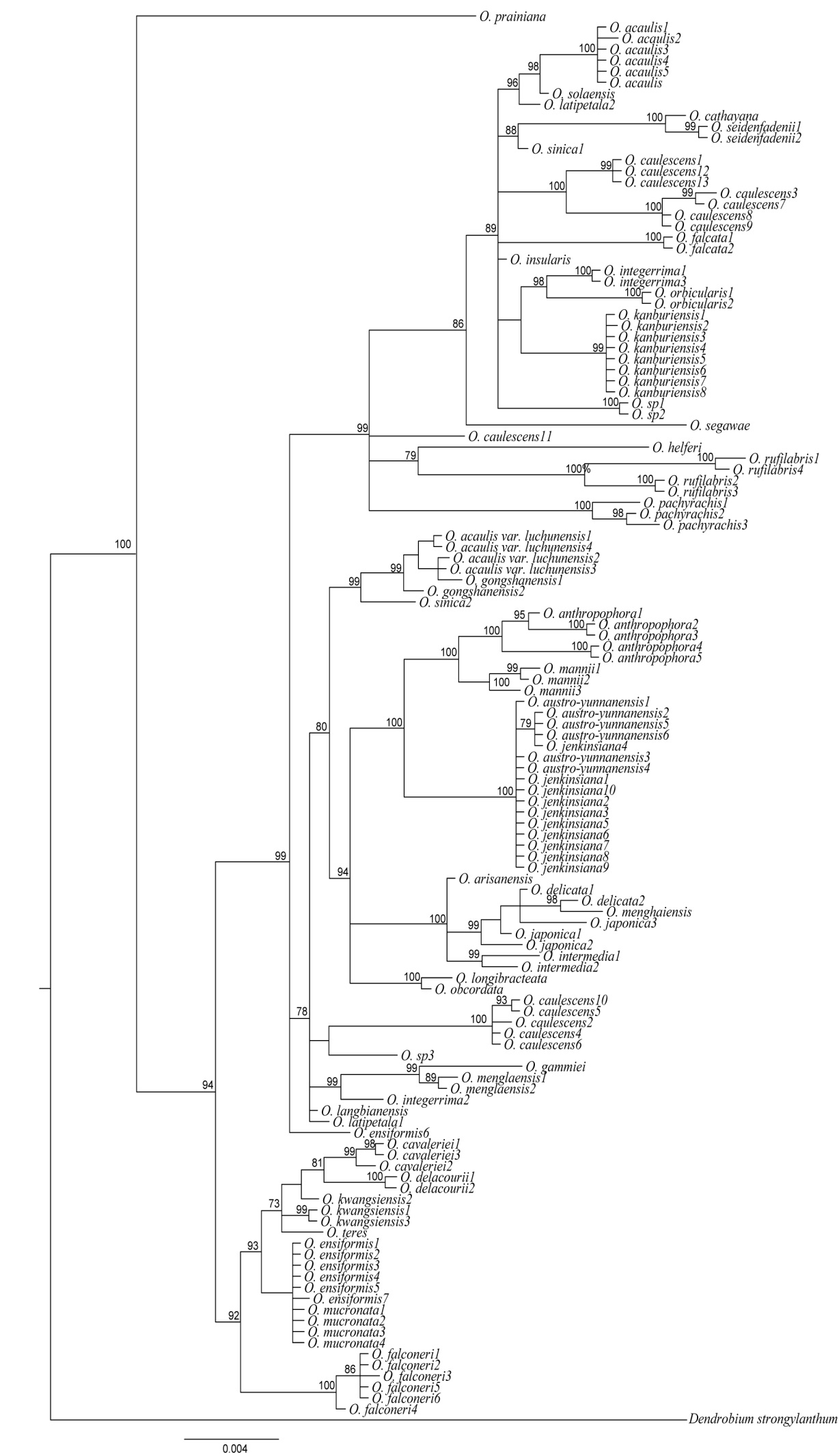
、

Fig. S3 (L): *matK+trnH*-*psbA*


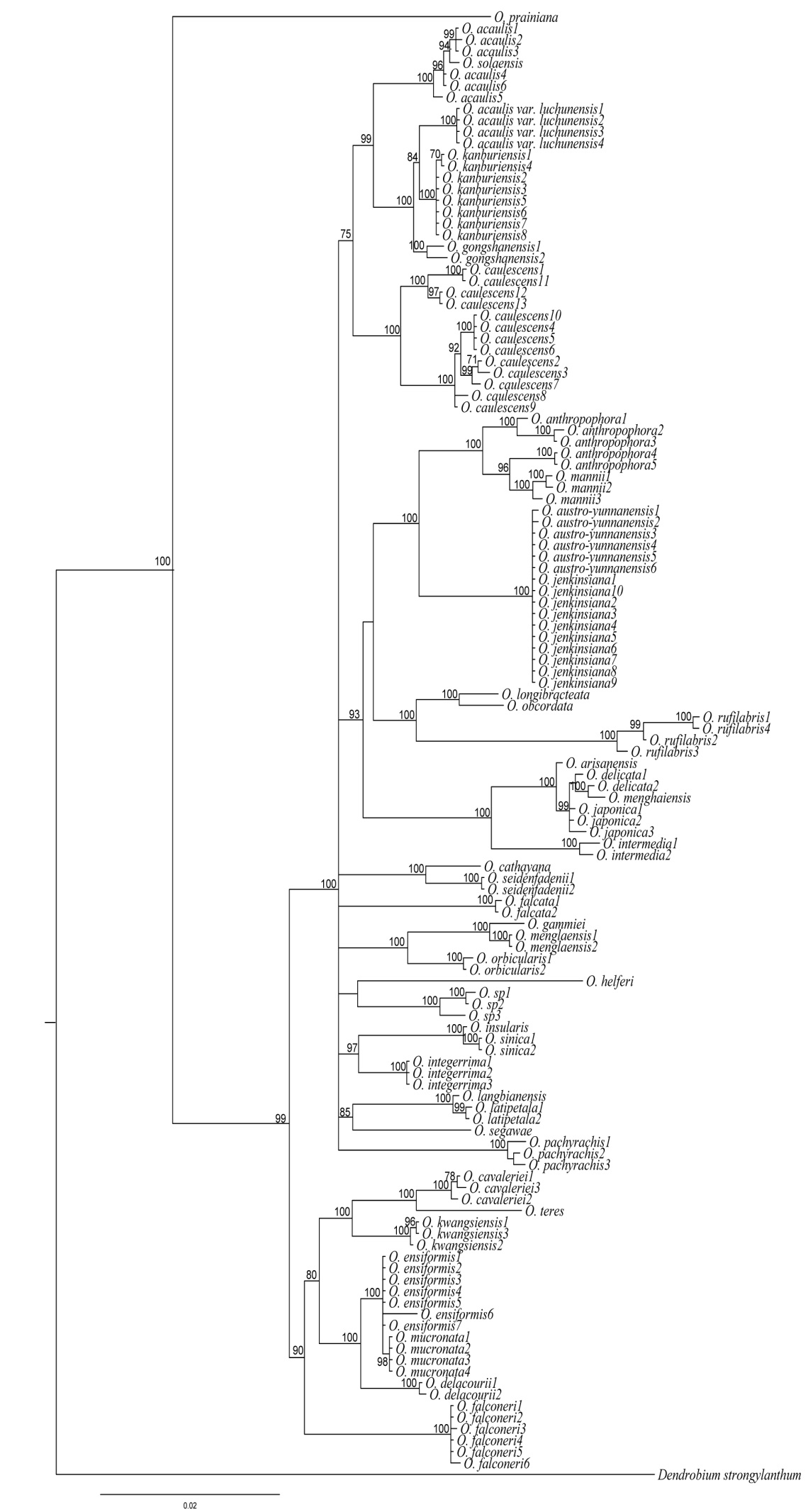


Fig. S3 (M): *rbcL+matK+*ITS


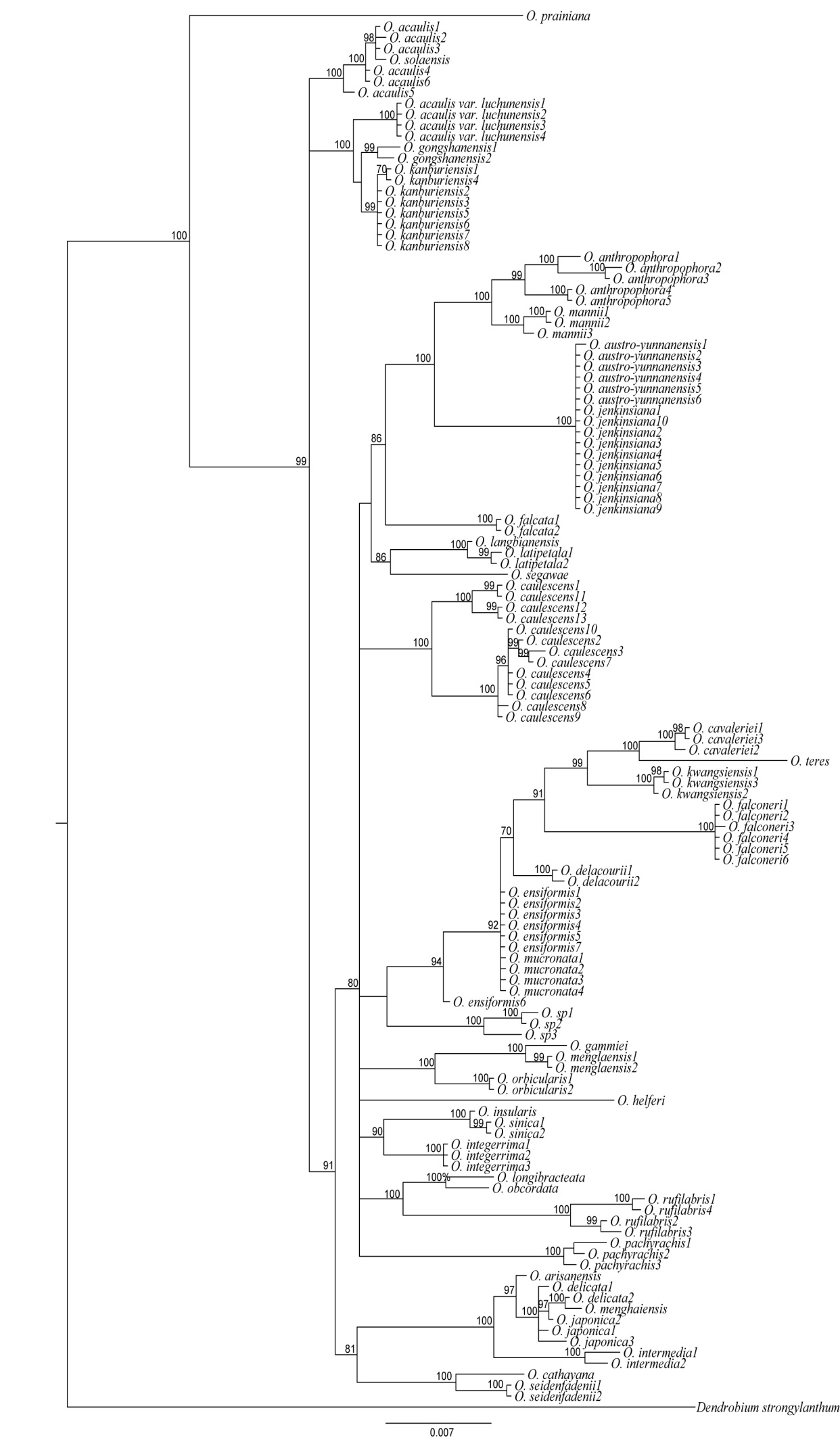


Fig. S3 (N): *rbcL+matK+*ITS2


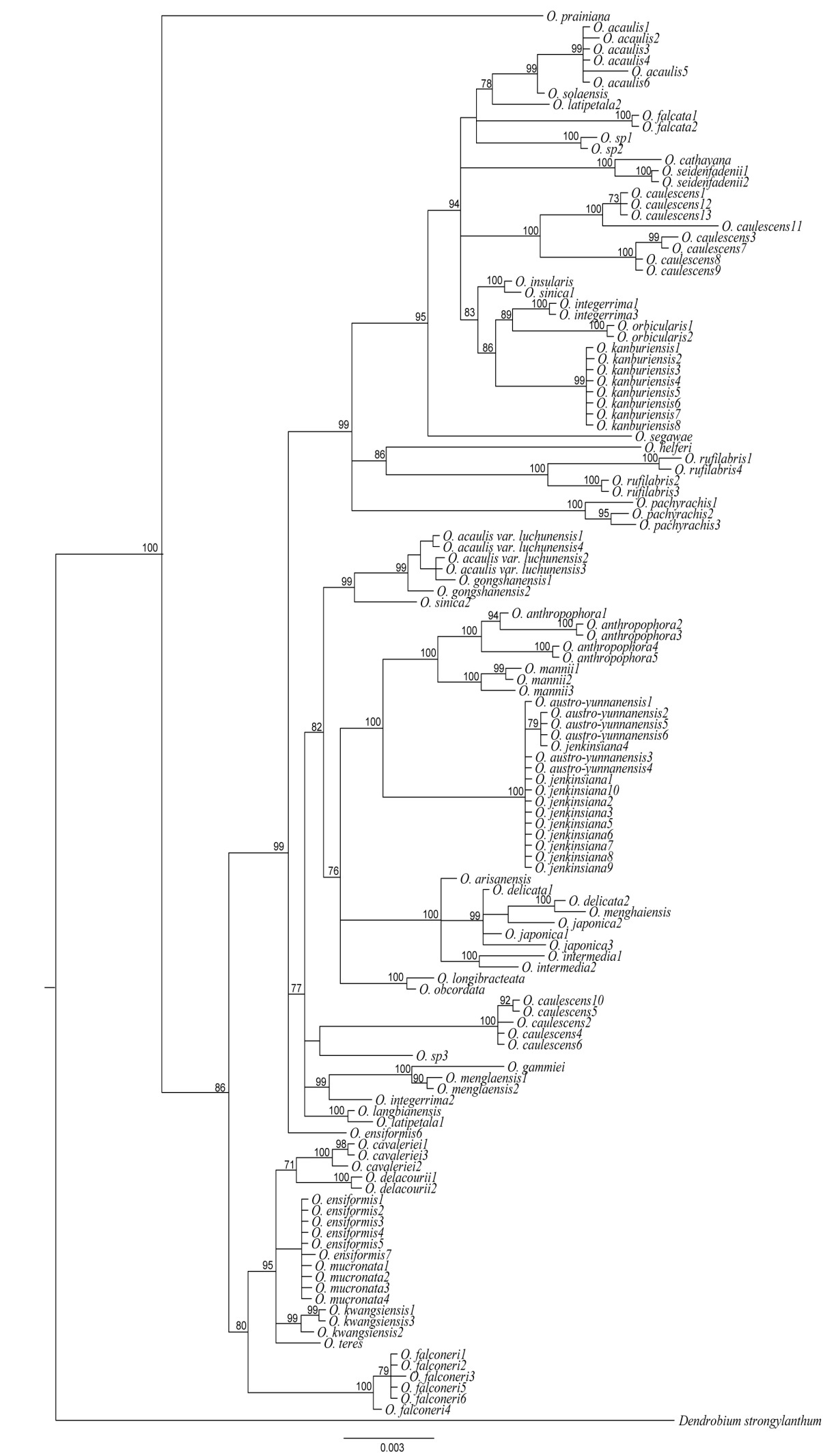


Fig. S3 (O): *rbcL+matK+trnH*-*psbA*


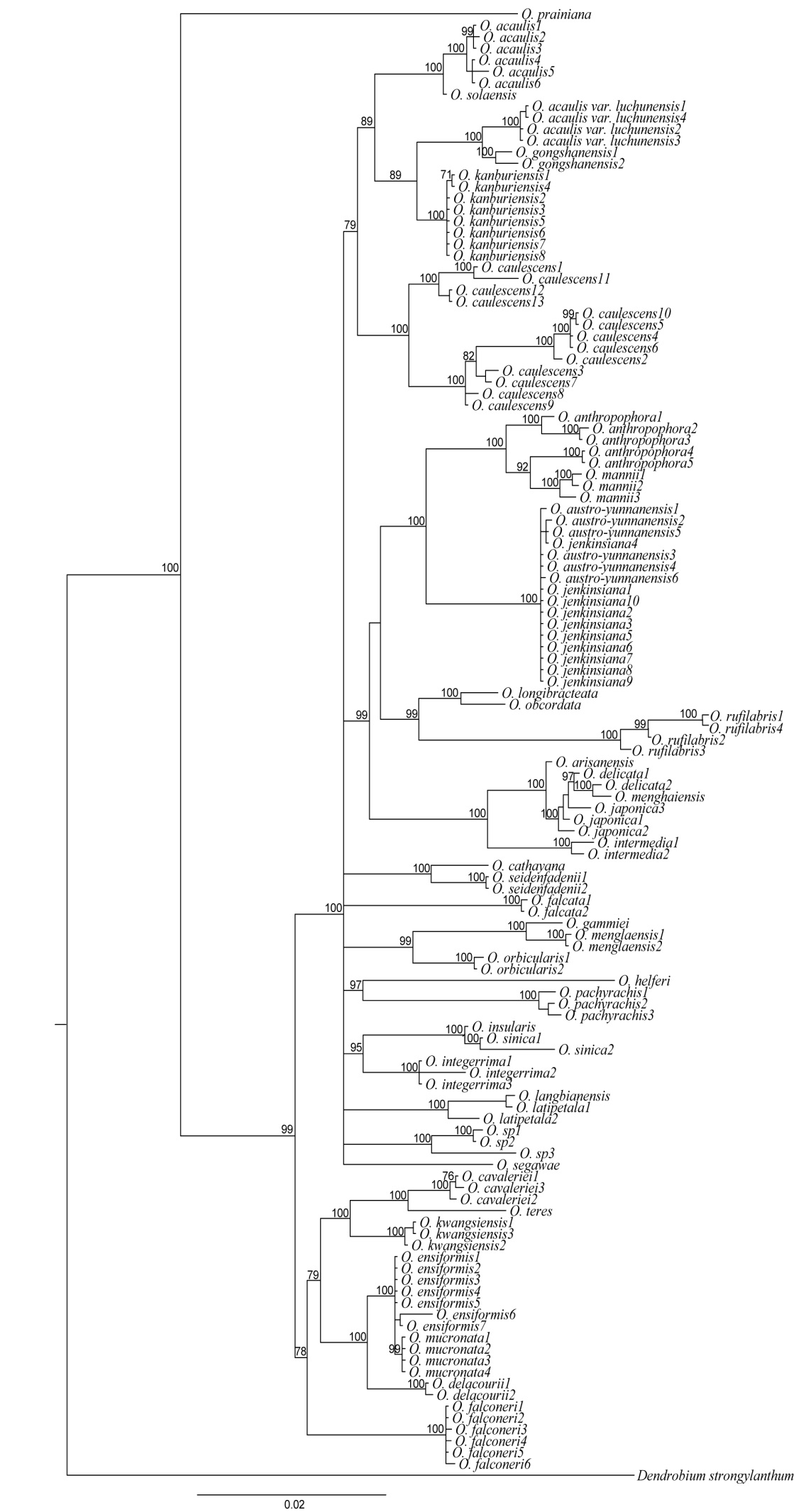


Fig. S3 (P): *rbcL+matK+*ITS*+trnH*-*psbA*
